# Ion transporter cascade, reactive astrogliosis and cerebrovascular diseases

**DOI:** 10.3389/fphar.2024.1374408

**Published:** 2024-04-09

**Authors:** Md Shamim Rahman, Rabia Islam, Mohammad Iqbal H. Bhuiyan

**Affiliations:** ^1^ Department of Pharmaceutical Sciences, School of Pharmacy, University of Texas at El Paso, El Paso, TX, United States; ^2^ Nostrum Hospital, Dhaka, Bangladesh

**Keywords:** reactive astrocytes, ion transporters, vascular dementia, VCID, Alzheimer’s disease, NKCC1, ZT-1a, BCAS

## Abstract

Cerebrovascular diseases and their sequalae, such as ischemic stroke, chronic cerebral hypoperfusion, and vascular dementia are significant contributors to adult disability and cognitive impairment in the modern world. Astrocytes are an integral part of the neurovascular unit in the CNS and play a pivotal role in CNS homeostasis, including ionic and p^H^ balance, neurotransmission, cerebral blood flow, and metabolism. Astrocytes respond to cerebral insults, inflammation, and diseases through unique molecular, morphological, and functional changes, collectively known as reactive astrogliosis. The function of reactive astrocytes has been a subject of debate. Initially, astrocytes were thought to primarily play a supportive role in maintaining the structure and function of the nervous system. However, recent studies suggest that reactive astrocytes may have both beneficial and detrimental effects. For example, in chronic cerebral hypoperfusion, reactive astrocytes can cause oligodendrocyte death and demyelination. In this review, we will summarize the (1) roles of ion transporter cascade in reactive astrogliosis, (2) role of reactive astrocytes in vascular dementia and related dementias, and (3) potential therapeutic approaches for dementing disorders targeting reactive astrocytes. Understanding the relationship between ion transporter cascade, reactive astrogliosis, and cerebrovascular diseases may reveal mechanisms and targets for the development of therapies for brain diseases associated with reactive astrogliosis.

## 1 Introduction

Astrocytes are crucial for brain health, supporting functions like neuron connectivity, blood flow regulation, energy provision, waste clearance, and chemical balance (Bélanger et al., 2011). Reactive astrocytes, activated in response to brain damage or disease or with aging, contribute to conditions like mild cognitive impairment and Alzheimer’s disease (Habib et al., 2020; Price et al., 2021). Additionally, vascular contributions to cognitive impairment and dementia (VCID) including Post-stroke dementia (PSD) are recognized as significant causes of dementia, often co-existing with Alzheimer’s disease (AD). Despite this recognition, the mechanisms of VCID remain poorly understood (Price, Johnson and Norris, 2021). Several studies from our lab and others implicated the role of several ion transporters in reactive astrogliosis and dementing disorders including VCID (Schaub and Schnitker, 1988; Bhuiyan et al., 2024a). This review explores the potential involvement of astrocytes in the development of VCID and AD and emphasizes reactive astrocytes as potential targets for developing broad therapeutic interventions.

### 1.1 Exploring reactive astrocytes and cerebrovascular involvement in VCID, with emphasis on PSD and AD

Cerebrovascular disease encompasses a range of medical conditions affecting blood circulation and the blood vessels in the brain, leading to disruptions in blood flow caused by conditions such as stenosis, thrombosis, embolism, or hemorrhage. Dementia is a syndrome characterized by a chronic or progressive decline in cognitive function, extending beyond normal aging effects (WHO, 2023). It impacts memory, thinking, orientation, comprehension, learning capacity, language, and judgment, often accompanied by changes in mood and behavior (WHO, 2021). Currently the seventh leading cause of death globally (WHO, 2023), dementia affected 55.2 million people in 2019, with projections estimating the number of dementia patients to be 78 million in 2030 and 139 million in 2050 (WHO, 2023). The associated global costs are anticipated to rise to US$1.7 trillion by 2030, reaching US$2.8 trillion when accounting for increased care expenses (Livingston et al., 2020; WHO, 2021). Numerous cerebrovascular diseases, stroke being one of the most studied, compromise the supply of blood to the brain, contributing to the development of dementia (Thacker et al., 2013; Iadecola et al., 2016; Gottesman et al., 2017). After Alzheimer’s disease (AD), cerebrovascular disease is the second most prevalent factor associated with cognitive disorders, commonly known as VCID (vascular contributions to cognitive impairment and dementia) (Corriveau et al., 2016), is estimated to contribute as the major or only etiological factor in 15%–25% cases and is often coexisting with AD (Gorelick et al., 2011; Oveisgharan et al., 2022). The World Health Organization has recently released the Blueprint for Dementia Research and highlighted VCID as a specific area that needs increased attention (WHO, 2017; Cataldi et al., 2023). With the aging of populations and an annual increase of 7.7 million new cases, the severity of the health crisis linked to dementia is approaching alarming levels, potentially giving rise to a “dementia epidemic” with profound socioeconomic consequences (Corriveau et al., 2016; Patterson, 2018; Iadecola et al., 2019). Due to substantial economic and health burdens, coupled with the absence of effective therapeutic interventions, there is a pressing demand for the advancement of treatments targeting VCID. Acknowledging the crucial role of reactive astrogliosis in cerebrovascular and dementing disorders, this review emphasizes the potential strategy of targeting astrocyte signaling for therapy development in disorders associated with VCID, specifically highlighting post-stroke dementia (PSD) and AD.

The clinical diagnostic criteria for VCID show some ambiguity, and there is a difference of preference regarding the usage of the terms VCI (Vascular Cognitive Impairment) and VCID (Gorelick et al., 2011; Hainsworth et al., 2021). VCID involves the neurovascular unit failing to cope with insults during aging, associated with systemic and cerebral vascular disease, proteinopathy, metabolic disease, and/or immune response, resulting in cognitive decline (Snyder et al., 2015). The primary feature of VCID is that cognitive impairments can be traced back to brain injury caused by cerebrovascular disease. VCID research spans various clinical diagnoses, encompassing cerebrovascular and cardiovascular diseases, stroke, AD, and other dementias from a vascular origin. Various pathophysiological processes for VCID associated with cerebrovascular disease have been identified through human brain imaging, post-mortem studies, and evidence from animal models (Iadecola et al., 2019; Hosoki et al., 2023).

Vascular Impairment of Cognition Classification Consensus Study (VICCCS) guideline defines PSD as a subcategory of VCID (Iadecola et al., 2019). PSD and post-stroke cognitive impairment (PSCI) impact around one-third of stroke survivors, predominantly within the initial 6 months. PSD is characterized not as a distinct disease but as an unspecified dementia syndrome that manifests after a stroke (Mijajlovic et al., 2017; Iadecola et al., 2019). Stepwise associations are observed between the severity of cerebrovascular events and both pre-event and post-event dementia, regardless of age, with the risk being affected by prior strokes, recurrent strokes, and markers of cerebral susceptibility (Pendlebury et al., 2019). Data from STROKOG (international cohorts in the Stroke and Cognition Consortium), a consortium of post-stroke/TIA (**transient ischemic attack)** or high vascular risk studies from around the world shows 44% of stroke survivors in hospital-based cohorts have global cognitive impairment (Lo et al., 2019). Stroke survivors experience faster cognitive decline in the first 1–3 years after onset compared to those without a stroke history (Lo et al., 2022). Diabetes is linked to poorer cognitive performance 3–6 months post-stroke (Lo et al., 2020). PSD is characterized by white matter lesions and reactive astrogliosis (Chen et al., 2016; Huang et al., 2020). PSD remain highly prevalent and disabling even after clinical recovery (Jokinen et al., 2015; Pendlebury, Rothwell and Oxford Vascular, 2019). Several acknowledged risk factors for poststroke dementia are potentially modifiable, suggesting that targeted interventions, therapeutics, or management strategies have the potential to reduce the risk of poststroke dementia (Rost et al., 2022). Reactive astrocytes in the glial scar are recognized as a major obstacle to neurite regeneration, hampering the overall functional recovery from a stroke. Gaining deeper insights into the mechanisms of reactive astrogliosis and glial scar formation could reveal new therapeutic targets aimed at fostering neurological functional recovery following a stroke and PSD (Liu and Chopp, 2016; Zhang et al., 2018; Huang et al., 2020).

Cerebral vascular pathology frequently coexists with AD pathology and plays a significant role in shaping the clinical cognitive profile of AD (Kalaria and Ballard, 1999; Iadecola, 2013). Epidemiological research suggests that the origins of PSD can be associated with AD or a blend of AD and vascular dementia in 29%–61% of individuals affected by PSD in developed countries (Leys et al., 2005). Within this framework, the “double-hit theory” proposes that experiencing severe cognitive impairment during a stroke may elevate the likelihood of developing dementia. This implies that an ischemic stroke could instigate supplementary pathophysiological mechanisms, potentially setting off a secondary degenerative pathway. This pathway may interact with the pathology of AD, hastening the progression of primary neurodegeneration (Thiel et al., 2014; Huang et al., 2020). Reactive astrocytes also contribute causatively to the pathophysiology and cognitive decline in AD (Chun and Lee, 2018; Price, Johnson and Norris, 2021). Alois Alzheimer, after whom the disease is named, first noticed the pathological changes in astrocytes within the brains of individuals with dementia, observing the abundant presence of glial cells within neuritic plaques (Alzheimer, 1910). Evidence from imaging studies indicates that astrogliosis could be an early occurrence in the disease (Arranz and De Strooper, 2019; Pelkmans et al., 2024) and this is further supported by biomarker studies (Mila-Aloma et al., 2020; Rodriguez-Giraldo et al., 2022). The characteristics of ‘reactive astrocytes’ appear to be influenced by different triggers, introducing uncertainties in predicting their phenotype solely from marker gene expression. The impact of AD-associated proteopathy (Aß and tau) on astrocytes remains an unresolved question (De Strooper and Karran, 2016; Arranz and De Strooper, 2019; Jiwaji et al., 2022). Given the critical role of reactive astrogliosis in different cerebrovascular and dementing disorders, targeting astrocyte signaling will be a potential viable strategy to develop therapy for dementing disorders like VCID associated with reactive astrogliosis, including PSD and AD. This review will mainly focus on how to target astrocyte signaling to develop therapy for dementing disorders.

## 2 Roles of astrocytes in the brain

Astrocytes are the most abundant and diverse glial cell types in the central nervous system (CNS) and play essential roles in the organization and maintenance of brain structure and function. As a key component of the neurovascular unit, astrocytes regulate cerebral blood flow through various mechanisms. Astrocytic endfeet wrap intraparenchymal blood vessels and release prostaglandins and nitric oxide which can either dilate or constrict blood vessels (Belanger and Magistretti, 2009). Other physiological functions of astrocytes include the maintenance of fluid, ion, pH, and neurotransmitters homeostasis in the extracellular space, promoting myelin sheath formation, synapse formation and maturation (Nishida and Okabe, 2007; Allen and Eroglu, 2017; Traiffort et al., 2020). Astrocytes removes excessive glutamate and helps to maintain normal neuronal activity and excitability (Mahmoud et al., 2019). Astrocytes serve as repositories for glycogen, acting as a reserve energy source in the brain. When faced with energy demand or low glucose levels, astrocytes metabolize glycogen into glucose, providing essential glucose and ATP to support normal brain function (Brown and Ransom, 2007; Magistretti and Allaman, 2015). Astrocytes enhance neuronal glucose uptake by increasing GLUT1 (glucose transporter type 1) activity (Simpson et al., 2007), with recent studies showing that astrocytic insulin signaling links to GLUT1 activity, fostering glucose uptake (Garcia-Caceres et al., 2016). Notably, astrocytic insulin receptor (*Insr*) ablation disrupts brain astroglial morphology and energy homeostasis (Garcia-Caceres et al., 2016; Gonzalez-Garcia et al., 2021). Additionally, astrocytes provide defense against oxidative stress via ROS-detoxifying enzymes (i.e., glutathione S-transferase, glutathione peroxidase, and catalase) to augment oxidative energy metabolism (Desagher et al., 1996; Dringen et al., 1999). Astrocytes also play compensatory role after ischemic stroke where astrocytes differentiated into neural progenitors and contributed to improved behavioral recovery (Li W et al., 2022). In the presence of astrocytes, neurons also show greater resistance to toxic doses of nitric oxide, hydrogen peroxide, superoxide anion or iron (Dringen et al., 1999; Belanger and Magistretti, 2009).

On the contrary, in response to CNS insults such as ischemia, infection or stress, astrocytes undergo a massive change in morphology, gene expression, and function, known as reactive astrogliosis (Escartin et al., 2019; Escartin et al., 2021). Although reactive astrogliosis are the hall mark of many metabolic and cerebrovascular disorders (Li T et al., 2019; Sofroniew, 2020), debate persists on whether the function of reactive astrocytes in conditions such as stroke and its aftermath (post-stroke neurodegeneration or PSD), along with other cognitive disorders like AD and related vascular dementias, is protective or cytotoxic. Based on their functional characteristics, reactive astrocytes can be categorized into two types, namely, inflammatory “A1” astrocyte and protective “A2” astrocyte (Liddelow et al., 2017; Sofroniew, 2020). It is important to recognize that A1/A2 paradigm is a straightforward and debatable classification method (Escartin, Guillemaud and Carrillo-de Sauvage, 2019) because several recent single-cell and single-nucleus RNA sequencing (sNuc-seq) studies reported many subpopulations of astrocytes with distinct gene expression profile (Guo et al., 2021; Hasel et al., 2021). For example, using sNuc-seq of the hippocampi of 7-month-old mice of either WT or a transgenic model of AD (5xFAD), Habib et al. found six clusters of astrocytes based on their transcriptional profiles (Habib et al., 2020). Notably, within the AD brain, they identified a distinct subset of astrocyte (cluster 4) with elevated levels of *Gfap*, termed disease-associated astrocytes (DAAs). These astrocytes appeared at early disease stages and increased in abundance with disease progression. Compared to WT astrocytes, those in AD exhibited increased expression of pan-reactive and inflammation/A1 astrocyte signatures but did not show an increase in A2 astrocyte signatures (Habib et al., 2020). Interestingly, most of the A1 signature genes were found to be expressed by DAAs, suggesting that DAAs and A1 astrocytes share similar neurodegenerative cascades including response to toxic compounds and inflammation. While further studies are warranted to fully understand the roles of various reactive astrocyte subpopulations, here we briefly highlight the role of “A1” and “A2” astrocytes in the context of cerebrovascular diseases.

### 2.1 Proinflammatory A1 astrocytes

A1/A1-like astrocytes, also known as proinflammatory or cytotoxic reactive astrocytes, show potent cytotoxicity by releasing a soluble toxin, precipitating in death of a subset of CNS neurons and oligodendrocytes (OL) (Liddelow et al., 2017). Co-culture with A1 astrocytes manifests significant neuronal death, underscoring their critical role on neurodegeneration. Induction of an A1-like state is frequently orchestrated by microglial-secreted cytokines, including interleukin-1 α (IL-1α), tumor necrosis factor α (TNFα), and complement component 1q (C1q) (Liddelow et al., 2017; Yun et al., 2018). Evidence suggests that M1 microglia activation triggers A1 astrocytes phenotype (Liddelow et al., 2017). Once activated, A1-like astrocytes deviate from their typical supportive functions, instead releasing factors deleterious to neighboring neurons and oligodendrocytes (Liddelow et al., 2017; Li X et al., 2020). Exploration of the A1 astrocyte transcriptome has identified 57 induced genes, with complement component 3 (C3) being a predominant marker (Zamanian et al., 2012). Several studies implicate A1-like and AD astrocytes in synaptic loss through the upregulation of complement component (C3 and C1q), synapse elimination through phagocytosis and synaptotoxicity due to impaired glutamate transport and signaling in neurodegenerative diseases including Alzheimer’s disease, amyotrophic lateral sclerosis (ALS), and multiple sclerosis (MS) (Wu et al., 2019; Dalakas et al., 2020; Hulshof et al., 2022). Additionally, investigations suggest that the upregulation of DAA and A1-like reactive genes in the aging brain may contribute to cognitive decline and heightened vulnerability to neurodegenerative processes (Clarke et al., 2018; Habib et al., 2020). The activation of NLRP3 inflammasome in microglia also serves as a trigger for the transformation of astrocytes into the A1 subtype (Xiao et al., 2022). In acute trauma, neuroinflammatory A1 reactive astrocytes may be induced by NF-κB signaling (Brambilla et al., 2005). Downregulation of STAT3 is also implicated in A1 astrocyte induction (Reichenbach et al., 2019). Noteworthy pathways contributing to A1 astrocyte activation include glutamate and ATP release, inflammatory mediator secretion (prostaglandin D2 and IFN-γ), and cytotoxin Lipocalin 2 (LCN2) secretion (Bi et al., 2013; Li K et al., 2019). This intricate network underscores the multifaceted nature of A1 astrocyte responses in various neurological contexts, from neurodegenerative diseases to acute injury, shedding light on potential therapeutic targets for modulating their functions.

### 2.2 Neuroprotective A2 astrocytes

A2 astrocytes, activated in response to brain injury or stroke, showcase a neuroprotective phenotype characterized by the upregulation of numerous neurotrophic factors such as BDNF, VEGF and bFGF (Li K et al., 2019). These factors play a pivotal role in promoting the survival and growth of neurons, as well as facilitating synapse repair through the increased expression of thrombospondins (Christopherson et al., 2005). Research highlights the diverse roles of A2 astrocytes in promoting CNS recovery and repair following ischemic stroke (Li S et al., 2022). Hyvärinen et al. showed, co-stimulation with microglial IL-1β and TNF-α produces A2 astrocyte phenotype, possibly induced by M2 microglia activation (Hyvarinen et al., 2019). Studies indicate that A2 astrocytes promote the expression of transforming growth factor β (TGF-β), contributing to axon formation and neuroprotection (Ruocco et al., 1999; Li T et al., 2020). The specific markers for identifying A2 astrocytes include S100 calcium-binding protein A10 (S100A10), a member of the S100 protein family, known for its roles in cell proliferation, membrane repair, and inhibition of apoptosis (Li T et al., 2019). In contrast to A1 astrocytes, A2 astrocytes do not express C3, making it an effective marker for distinguishing between the two phenotypes (Li T et al., 2019; Fei et al., 2022). Studies involving selective ablation of scar-forming reactive astrocytes, marked by the STAT3 activation, underscore the importance of these cells in preventing excessive inflammation and maintaining blood-brain barrier integrity (Sofroniew, 2015). Because A2 astrocytes contribute to survival, growth, and repair of neurons and OLs, strategies that promote the formation of A2 astrocytes could be a promising approach for treating cerebrovascular injuries and PSD.

## 3 Ion transporter cascade central to reactive astrogliosis

**FIGURE 1:**
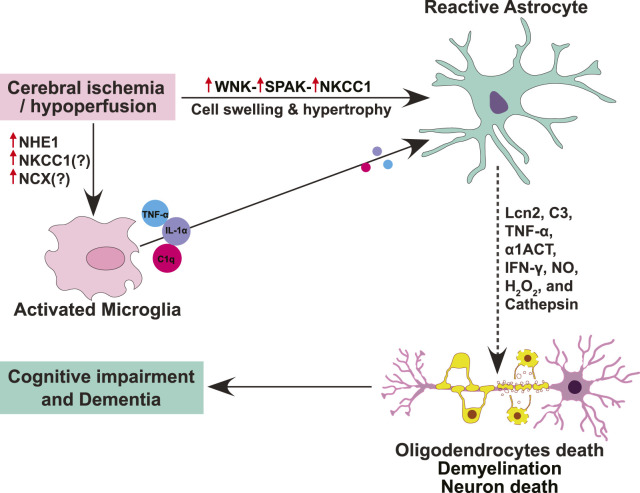
Schematic summary of ion transporter signaling involved in reactive astrogliosis, oligodendrocytes death, and cognitive impairment. Cerebral ischemia or hypoperfusion triggers upregulation and activation of astrocytic WNK-SPAK-NKCC1 cascade proteins, causing intracellular Na^+^ overload, cell swelling and hypertrophy, and ultimately leading to astrogliosis. Different cytotoxic molecules (Lcn2, C3, TNF-α, α1ACT, IFN-γ, NO, H_2_O_2_, and cathepsin) secreted by reactive astrocytes mediate the death of oligodendrocytes and neurons, leading to demyelination and cognitive impairment. Moreover, ischemia-induced activation of NHE1 contributes to microglial activation which subsequently induces reactive astrogliosis through the secretion of pro-inflammatory cytokine cocktails (TNF-α, IL-1α, and C1q).

Upon insult or injury, reactive astrogliosis response is orchestrated by intricate signaling cascades that often involve ion channels and transporters (McConnell et al., 2019). At the forefront of this dynamic process are ion channels such as aquaporins, transient receptor potential (TRP) channels, Ca^2+^-activated K^+^ (KCa) channels, ATP-sensitive K^+^ (K-ATP) channels, and ion cotransporters including Na^+^-K^+^-2Cl^-^ cotransporter 1 (NKCC1) and sodium-hydrogen exchanger isoform 1 (NHE1) (Figure 1). The initiation of reactive astrogliosis often stems from disruptions in ion homeostasis, particularly involving Ca^2+^, K^+^, and water flux (Song et al., 2019). Aquaporin-4 (AQP4), a pivotal water channel highly expressed in astrocytes, plays a dual role in lymphatic drainage and interstitial fluid exchange. Its deficiency impedes the clearance of solutes, contributing to astrocytic swelling and cerebral edema. TRP channels, sensitive to various stimuli, mediate Ca^2+^ influx, crucial for astrocytic function (Wang et al., 2022; Zhou et al., 2022). However, under pathological conditions, TRP channels are activated by reactive oxygen species (ROS), inflammatory factors, leading to Ca^2+^ dysregulation and astrocytic Ca^2+^ overload (Shigetomi et al., 2013; Wang et al., 2022). KCa channels, notably KCa3.1, respond to intracellular Ca^2+^ levels, participating in membrane potential regulation. In reactive astrogliosis, KCa3.1 activation elevates pro-inflammatory factors, linking ion dysregulation to neuroinflammation. K-ATP channels, sensitive to ATP levels, contribute to astrocyte-mediated glutamate uptake and spatial K^+^ buffering, disruptions of which are implicated in neurodegenerative diseases.

Ion transporters cascades also play a vital role in regulating the intra- and extra-cellular p^H^, Na^+^, K^+^, and Ca^2+^ homeostasis and astrocyte function. However, when overstimulated, ion transporters can contribute to inflammation, excitotoxicity, and reactive astrogliosis, contributing to cerebrovascular diseases (Boscia et al., 2016). The ion transporters within astrocytes orchestrates a complex cascade central process of astrogliosis following CNS insults. Ion transporter NKCC1, NHE1, sodium-calcium exchanger (NCX), and sodium-bicarbonate cotransporter (NBC) are known to be involved in reactive astrogliosis following CNS insults or stress (Annunziato et al., 2013). Rapidly activated by cytokines in the ischemic penumbra, astrocytes transform into reactive states and form glial scars. The delicate balance between detrimental and restorative functions of reactive astrocytes hinges on the activities of these ion transporters. NHE1, ubiquitously expressed in the central nervous system, regulates p^H^ and cell volume homeostasis. Its activation during ischemia contributes to astrocytic swelling and the release of proinflammatory cytokines, yet its inhibition showcases promise in reducing brain edema and inflammation for tissue repair. NCX, in both forward and reverse modes, emerges as a key player in synaptic plasticity, excitotoxicity, ER stress, and astrocyte apoptosis. NKCC1, dominant in the brain, is pivotal for cell volume regulation and excitotoxicity. Interestingly, studies from our lab and others found that NKCC1 activity causes astrocytic intracellular Na^+^ overload, hypertrophy, and swelling, and leads to astrogliosis after *in vitro* and *in vivo* ischemia or ammonia-induced toxicity (Su et al., 2002; Rangroo Thrane et al., 2013). This highlights NKCC1 protein as a potential therapeutic target for various CNS diseases associated with reactive astrogliosis including VCID, PSD and AD. Moreover, NKCC1 activity is regulated by two downstream regulatory kinases–WNK [“with no lysine” (K)] kinase and SPAK/OSR1 (Ste20/SPS1-related proline/alanine-rich kinase and oxidative stress-responsive kinase 1) kinases (Richardson et al., 2008; Zhang et al., 2020). The WNK and SPAK kinases are involved in multiple neurological disorders (Begum et al., 2015; Karimy et al., 2017; Zhao et al., 2017).

Hence, similar to NKCC1, WNK-SPAK kinases emerge as promising drug targets for addressing vascular-origin dementing illnesses. This highlights a novel method of intervention for neurological changes in cerebrovascular diseases by targeting and modulating reactive astrocyte signaling through the regulation of NKCC1, WNK-SPAK kinases. Developing approaches to regulate astrocyte reactivity, whether by restoring homeostatic functions or promoting a neuroprotective phenotype, may offer a new strategy to mitigate neuroinflammation and, consequently, impede the progression of dementia in conditions such as AD, PSD, and other forms of VCID.

## 4 Therapeutic strategies to attenuate reactive astrogliosis and associated cerebrovascular diseases

An emphasis in recent research has been on the proactive management of 12 modifiable risk factors, outlined by the World Health Organization in their guidelines for reducing the risk of cognitive decline and dementia (Livingston et al., 2020). This offers optimism that early intervention in areas such as astrogliosis could potentially reverse dementia entirely. Here we have summarized potential therapeutic strategies (Table 1) to attenuate reactive astrogliosis and associated neurological changes in cerebrovascular diseases.

**TABLE 1:** Potential therapeutic agents for cerebrovascular diseases.

Drug class	Drug name	Disease/model	Target	Function/outcome	References	Clinical trial
**Ion transporter cascade inhibitors**	Bumetanide (BTN or BMT or BUM)	Ischemic Stroke, AD, VCID, Neonatal seizures, Autism	NKCC1 and NKCC2	• Potent loop diuretic	Bhuiyan et al., 2017; Boyarko et al., 2023; Graber-Naidich et al., 2023a, Graber-Naidich et al., 2023b; Kharod et al., 2019; O'Donnell et al., 2004; Savardi et al., 2021; Sivakumaran and Maguire, 2016; Taubes et al., 2021; Yu et al., 2018	FDA-approved potent loop diuretic
				• Decrease acute ischemia- or chronic hypoperfusion-induced white matter damage, astrogliosis, brain edema and infarction		Drug candidate for treating APOE4-related AD
				• Improves neurological and cognitive functions		Phase IIa (NCT06052163)
	BUM13 (Bumepamine, lipophilic benzylamine derivative of bumetanide)	Epilepsy and Kidney diseases	NKCC2A	• Penetrate the BBB	Brandt et al., 2018; Lykke et al., 2015; Romermann et al., 2017	Preclinical
				• Inhibit NKCC2A		
				• Potentiate the anti-seizure effect of phenobarbital		
	STS5/BUM5 (N,N-dimethylaminoethylester of bumetanide/DIMAEB)	Ischemic stroke, Epilepsy and Kidney diseases	NKCC1	• Reduce ischemic infarction and cerebral edema	Auer et al., 2020; Boyarko et al., 2023; Erker et al., 2016; Huang et al., 2019; Johne et al., 2021; Romermann et al., 2017; Tollner et al., 2014	Preclinical
				• Improve cognitive function		
				• Penetrate the BBB		
				• Potentiate the anti-seizure effect of phenobarbital		
	STS66	Ischemic stroke	NKCC1	Reduce stroke-induced hemisphere swelling, infarction and improve neurological functions	Huang et al. (2019)	Preclinical
	ZT-1a	Ischemic Stroke PSD, VCID	SPAK	• Reduce stroke-induced infarction, swelling, white matter lesions and improve neurological functions	Bhuiyan et al., 2024a; Bhuiyan et al., 2022; Zhang et al., 2020	Preclinical
				• Reduce astrogliosis, oligodendrocyte death, WML and improve cognitive functions		
				• Reduce C3d + A1 cytotoxic reactive astrocytes while preserving S100A10+GFAP + homeostatic A2 astrocytes		
	ZT-1a derivatives (1c, 1day, 1g and 1h)	Ischemic Stroke and PSD	SPAK	• Decrease ischemic brain lesion	Bhuiyan et al. (2023)	Preclinical
				• Preserve white matter integrity		
				• Improve neurological outcome		
	Closantel	Ischemic Stroke	SPAK	• Reduce stroke-induced infarction and hemisphere swelling	Gloeckner et al., 2010; Zhang et al., 2020	FDA-approved broad-spectrum salicylanilide veterinary antiparasitic drug for a variety of types of animals Preclinical (Ischemic stroke)
	WNK463	Ischemic Stroke, hypertension	WNK	• Reduce blood pressure and regulates body fluid and electrolyte homeostasis	Yamada et al., 2016; Zhang et al., 2020	Preclinicalß
				• No effect on stroke outcome		
	HOE642 (Cariporide)	Ischemic Stroke, VCID	NHE1	• Attenuate astrogliosis, microglial activation, and demyelination	Liu et al., 2021; Metwally et al., 2023	Preclinical
				• Improve white matter integrity and cognitive function		
				• Reduce stroke-induced brain infarction, swelling and improve neurological functions		
**GLP-1R agonists**	NLY01	PD and AD	Inhibits microglial secretion of inflammatory cytokines (IL-1α, TNFα, and C1q) cocktails	• Penetrate CNS and block pathologic α-synuclein-induced microglial activation	Park et al., 2021; Yun et al., 2018	Phase I trial (NCT03672604)
				• Protect against dopaminergic neuronal loss and cognitive deficits		NLY01-PD-1 (NCT04154072)
				• Attenuate A1 astrogliosis		Phase IIB Alzheimer’s study
				• Reduce pathologic oligomeric Aβ1-42-induced microglia activation		
	Semaglutide	Ischemic stroke and Dementia	Inhibits microglial secretion of inflammatory cytokines (IL-1α, TNFα, and C1q) cocktails	• Attenuate Iba-1+ microglia/macrophages, and C3d+/GFAP + A1 reactive astrocytes	Buie et al., 2019; Zhang et al., 2022; Zlokovic et al., 2020	FDA approved GLP-1R agonist for T2D (EVOKE, NCT04777396 and EVOKE Plus, NCT0477740)
				• Reduce BBB damage, brain infarction, and improve neurological function		Phase III (SELECT NCT0357459)
**NLRP3 inflammasome inhibitor**	MCC950	Experimental autoimmune encephalomyelitis (EAE)	NLRP3	• Reduce astrogliosis	Hou et al., 2023; Hou et al., 2020	Preclinical
				• Prevent EAE-induced demyelination		
				• Prevent transformation of cytotoxic A1 astrocytes and enhance protective A2 astrocytes		
				• Block microglial conversion to M1 microglia		
	JC-124	Traumatic brain injury (TBI) and AD	NLRP3	• Reduce number of Iba-1+ microglia/macrophages and Aβ deposition	Kuwar et al., 2019; Yin et al., 2018	Preclinical
				• Decrease brain inflammation		
				• Attenuate expression of IL-1β, TNF-alpha, and iNOs		
	OLT1177	AD	NLRP3	Reduce microglial activation and improves cognitive deficits	Lonnemann et al. (2020)	Preclinical
	RRx-001 (Nibrozetone)	AD and PD	NLRP3	• Penetrate blood-brain barrier	Jayabalan et al., 2023; Oronsky et al., 2023	Phase I (BRAINSTORM NCT02215512)
				• Reduce chronic inflammation		
	Minocycline	Early brain injury (EBI) and ischemic stroke	NLRP3	• Reduce the number of Iba-1+ microglial cells, IL-1β expression, and improve neurological functions	Hayakawa et al., 2008; Li et al., 2016; Lu et al., 2016; Sheng et al., 2018	Phase II (NCT05367362)
				• Reduce IL-1β and IL-18 cytokines level and cerebral infract volume		
**Inflammatory cytokine inhibitors and antibodies**	Rilonacept	Rheumatoid arthritis	IL-1 blocker	Reduce inflammation	Arnold et al. (2022)	FDA approved
	Anakinra	Rheumatoid arthritis and stroke	IL-1R antagonist	Reduce secondary brain damage following spontaneous hemorrhagic stroke	Arnold, Yalamanoglu and Boyman (2022)	FDA approved Phase II “ACTION” (NCT04834388)
	Canakinumab (IL-1β neutralizing antibody)	rheumatoid arthritis	IL-1β neutralizing antibody	Reduce inflammation	Arnold, Yalamanoglu and Boyman (2022)	FDA approved
	IL-1R blocking antibody (anti-IL-1R)	AD	IL-1R blocking	• Alleviate cognitive deficits and markedly attenuates tau pathology	Kitazawa et al. (2011)	Preclinical
				• Inhibit amyloid-β formation by decreasing NF-κB transcriptional activity		
				• Inhibit inflammatory A1 astrocyte		
	anti–IL-18 IgG antibody	Rat model of vascular injury	Endogenous IL-18 neutralization	Inhibit cytokine production and NF-κB activation	Maffia et al. (2006)	Preclinical
	anti-C1q antibody (humanized anti-C1q antibody: ANX005)	LPS-induced neuroinflammation	C1q-neutralization	Reduce microglia-dependent synaptic loss and cognitive impairments	Lansita et al., 2017; Wu et al., 2023	Phase I
	anti- TNFα antibody	Focal ischemic injury	TNFα neutralization	Provide neuroprotection against focal ischemic injury	Barone et al. (1997)	Preclinical
	Etanercept	AD	TNF-α inhibitors	Reduce activation of microglia and tau deposition	Ou et al. (2021)	Preclinical
	type-II human TNF-receptor to a transferrin receptor antibody	AD	TNFα neutralization	Reduce activation of microglia and tau deposition	Ou et al. (2021)	Preclinical
	Adalimumab	AD and VCID	TNF-α inhibition	• Reduce AD pathology and neurotoxicity by inhibition of NF-κB and improve cognitive deficits	Park et al., 2019; Xu H et al., 2021	Preclinical
				• Decrease microglial activation		
	Aducanumab (Human monoclonal antibody Aduhelm)	Cerebral amyloid angiopathy (CAA)	Neutralizes oligomeric parenchymal forms of Aβ	Clear Aβ deposits from brains by recruiting Iba1-positive microglia and GFAP-positive astrocyte to Aβ plaques	Kong et al., 2022; Loomis et al., 2024; Ries and Sastre, 2016; Sevigny et al., 2016	Accelerated FDA approved for AD (To be discontinued from 2024)
	Lecanemab (Humanized IgG1 antibody)	Early-stage AD	Aβ plaque	Reduce cognitive decline in early AD patients and Aβ plaque clearance	Prillaman, 2022; van Dyck et al., 2023	Phase III (NCT03887455, NCT05925621, NCT04468659, NCT01767311, NCT05269394)
	Gantenerumab (anti-Aβ IgG1 monoclonal antibody)	Early-stage AD	Aggregated Aβ plaque	Reduce amyloid plaque in early AD (Did not show a significant impact on slowing clinical decline compared to a placebo at 116 weeks)	Bateman et al. (2023)	Phase III (NCT03444870, NCT03443973)
	Solanezumab	AD	Binds to the central epitope of monomeric amyloid-β and inhibits nucleation site for Aβ oligomerization	Increase peripheral elimination and solubilize amyloid-β in the cerebrospinal fluid to reestablish equilibrium (Did not demonstrate a reduction in cognitive decline compared to a placebo over a 240-week period in those with preclinical AD)	Sperling et al. (2023)	Phase III (NCT02008357)
**Choline-containing phospholipids**	α-GPC	VCID, AD, and stroke	Transglutaminase	Improve learning and memory	Bramanti et al., 2008; Jeon et al., 2023; Parker et al., 2022; Salvadori et al., 2021; Tayebati et al., 2009	FDA registered drug or nutraceutical (NCT05050604)

### 4.1 Ion transporter cascade inhibitors

Since multiple ion transporters and their upstream regulatory kinases play critical roles in reactive astrogliosis process, pharmacological inhibitors for those proteins and kinases represent as promising drugs for cerebrovascular and neurodegenerative diseases.

One such inhibitor is the specific NKCC1 inhibitor, Bumetanide that shows promise as a potential therapy for AD (Taubes et al., 2021; Graber-Naidich et al., 2023a; Graber-Naidich et al., 2023b; Boyarko et al., 2023) and for modifying post-stroke brain changes (O'Donnell et al., 2004). Recently, Bumetanide has emerged as a leading repurposed FDA-approved drug candidate for treating APOE4-related AD (Taubes et al., 2021; Graber-Naidich et al., 2023a). A computational analysis with the Connectivity Map database identified Bumetanide as the top-scoring drug capable of reversing the APOE4-specific transcriptomic signatures of AD. Bumetanide treatment of human APOE4 knock-in (KI) mice (homozygous APOE4/APOE4) exhibited improved memory function, as assessed by the Morris water maze (MWM) test, and reduced brain electrophysiological and pathological deficits. In J20/APOE4 mice (APPFAD, human APP-751/770-Swe-Ind), Bumetanide treatment reduced Aβ plaque accumulation and rescued hyperactivity (Taubes et al., 2021). Human studies reveal a significant link between Bumetanide exposure and a lowered prevalence of AD in individuals aged 65 and above. Data from two electronic health record databases (Taubes et al., 2021) support this association, indicating Bumetanide’s potential as a therapeutic agent for AD and various dementias. A recent Phase IIa clinical trial has been initiated to assess Bumetanide’s safety and tolerability in confirmed AD patients, while also investigating its efficacy in those with mild cognitive impairment or mild dementia due to AD (Clinical Trials.gov Identifier: NCT06052163).

In a mouse model of VCID, Bumetanide attenuated chronic hypoperfusion-induced white matter damage and cognitive impairment (Yu et al., 2018). The administration of Bumetanide following bilateral carotid artery stenosis (BCAS) surgery did not visibly impact astrocytic and microglial reactivity. Nevertheless, Bumetanide administration promoted the proliferation of OPCs, increased cell densities in the oligodendrocyte lineage, facilitated oligodendrocyte proliferation and differentiation, evident in the heightened number of NG2-positive OPCs and myelination by GST-π-expressing oligodendrocytes in the corpus callosum (Yu et al., 2018). This action mitigated white matter damage induced by chronic hypoperfusion and ameliorated working memory impairment resulting from BCAS (Yu et al., 2018). These findings may have indirect implications for astrocyte reactivity through mechanisms of crosstalk between astrocytes and oligodendrocytes (Nutma et al., 2020). Moreover, many studies showed Bumetanide treatment significantly improved neurological and cognitive function in rodent model of ischemic stroke and brain injury (Bhuiyan et al., 2017; O'Donnell et al., 2004). Bumetanide treatment significantly decreased astrogliosis, brain edema and infarction. To improve BBB permeability, several lipophilic Bumetanide prodrugs including BUM5, BUM13, STS66, have recently been developed (Tollner et al., 2014; Romermann et al., 2017; Huang et al., 2019). Post-stroke administration of the NKCC1 inhibitor STS66 has been reported to attenuate astrogliosis, brain edema, and infarction, while improving neurological function after ischemic stroke. This suggests that Bumetanide and its prodrugs hold therapeutic potential for cerebrovascular and dementing disorders. Notably, the novel NKCC1 inhibitor STS66 surpasses Bumetanide and STS5 in reducing ischemic infarction, swelling, and neurological deficits in large-vessel transient ischemic stroke and permanent focal ischemic stroke with hypertension comorbidity (Huang et al., 2019).

NKCC1 activity is regulated by its upstream regulatory kinases–WNK and SPAK/OSR1 (Vitari et al., 2006). Recently we have developed a specific SPAK inhibitor, ZT-1a (Zhang et al., 2020) which can efficiently enter ischemic brain. Post-stroke administration of ZT-1a showed inhibitory effect on WNK-SPAK-NKCC1 signaling cascade leading to neuroprotective outcome including reduction of cerebral infarct volume, swelling, white matter lesions and improvement of neurological function after ischemia (Zhang et al., 2020; Bhuiyan et al., 2022), suggesting ZT-1a has therapeutic potential against PSD. Indeed, our very recent study found that chronic administration of ZT-1a attenuates the WNK-SPAK-NKCC1 signaling, prevents BCAS-induced WML and cognitive impairment in a mouse model of VCID (Bhuiyan et al., 2024b). We found that post-BCAS administration of ZT-1a (5 mg/kg, i. p) in C57BL/6J male mice prevented OL cell death and improves learning and memory function assessed by MWM and novel object recognition tests. Importantly, ZT-1a treatment significantly decreased reactive astrogliosis, increased oligodendrocytes differentiation and survival. Moreover, ZT-1a increased S100A10^+^GFAP^+^ protective astrocytes while reducing C3d^+^ A1 cytotoxic astrocytes in white matter tracts of mouse brains (Bhuiyan et al., 2024a). These findings underscore the therapeutic potential of ZT-1a in ameliorating the detrimental effects of cytotoxic reactive astrocytes in VCID and other forms of dementia.

The ion transporter NHE1 is pivotal in activating microglia and inducing reactive astrogliosis in various brain diseases, including several conditions implicated in the development of VCID (Liu et al., 2021; Luo et al., 2021). Genetic ablation of *Nhe1* resulted in reduced reactive astrogliosis, microglial activation, cerebral infraction, white matter lesions, and improved neurological function in mouse model of ischemic stroke (Begum et al., 2018; Song et al., 2018). This exhibits NHE1 as a therapeutic target for cerebrovascular and dementing disorders. Interestingly, recent studies from our lab showed that pharmacological inhibition of NHE1 with potent HOE642 inhibitor (Cariporide) and Rimeporide improved white matter integrity and cognitive function by reducing reactive astrogliosis in mouse models of VCID and ischemic stroke (Liu et al., 2021; Metwally et al., 2023). HOE642 treatment prevented demyelination by increasing myelin forming cells, oligodendrocytes and maintained white matter microstructure as evidenced in mouse brain with MRI imaging. HOE642 also reduced astrogliosis and microgliosis confirmed by lower count of GFAP^+^ and Iba1^+^ cells co-localization NHE1 protein in corpus callosum and external capsule of mouse brain. The application of HOE642 resulted in a downregulation of inflammatory genes (*Lcn2, Il1r1, IL-1β, IL-6, TNF-α, Il2rg, Tnfsf8, Mmp9, Cxcr2, Ccl5,* and *Itgal*) as well as reduction in both pan-reactive (*Vim, Lcn2, Cd44, Cxcl10, Hspb1, Gfap, Serpina3n, Osmr, and Timp1*) and A1 (*Gbp2, H2-D1, C3, C4b, Psmb8, Serping1, and Srgn*) astrocyte markers (Liu et al., 2021). These studies demonstrate that HOE642 can attenuate microgliosis and astrogliosis, most probably inflammatory A1 reactive astrocytes, a common pathology of AD, PSD, and several other vascular dementias. However, the mechanism of inhibition of A1 reactive astrocytes by HOE642, either by M1 microgliosis inhibition which has been shown to increase A1 reactive astrocytes or direct inhibition of A1 reactive astrocytes formation remains elusive (Park et al., 2021). The effect of HOE642 in AD and PSD remains poorly understood. Therefore, additional research is necessary to investigate the potential of HOE642 in inhibiting A1 reactive astrocytes and combating neurodegenerative diseases such as AD, PD, PSD, or related vascular dementias.

Collectively, inhibitors of ion transporters and their regulatory kinases demonstrate promise as potential therapeutic agents for treating cerebrovascular and dementing disorders. Nonetheless, future research should delve into the development of ion transporter inhibitors and their signaling inhibitors with favorable pharmacokinetic characteristics and enhanced blood-brain barrier permeability for clinical applications in dementia treatment.

### 4.2 GLP-1R agonist

Glucagon-like peptide-1 (GLP-1) hormone secreted from intestinal cells induces insulin secretion from pancreatic β cells upon binding to the GLP-1 receptor (GLP-1R) on the surface of β cells (Klegeris, 2021). GLP-1 can freely cross the blood-brain barrier and can regulate many neuroendocrine functions including learning and memory, rewards behavior, and reduces hunger-driven feeding, food’s hedonic value, and motivation along with its known incretin effect in hyperglycemic conditions (Diz-Chaves et al., 2020). Interestingly, reduced activity of GLP-1R in the CNS results in microglia activation and reactive astrogliosis (Park et al., 2021), suggesting that activation of GLP-1R by GLP-1R agonist could be a viable strategy to attenuate activation of microglia and astrogliosis in neurodegenerative diseases.

Given that GLP-1R agonists are presently employed in treating type 2 diabetes, their application for testing in VCID and AD could be swiftly implemented. Aligning with this hypothesis, several GLP-1R agonists have shown neuroprotective efficacy against AD and Parkinson’s disease (PD) pathology, reducing microgliosis and astrogliosis (Aviles-Olmos et al., 2013; Athauda et al., 2017; Park et al., 2021). For example, a pegylated exendin-4 GLP-1R agonist, NLY01, penetrates CNS and blocks pathologic α-synuclein-induced microglial activation both *in vitro* and *in vivo*, inhibits secretion of inflammatory cytokines IL-1α, TNFα, and C1q, in turn prevents reactive astrogliosis in human A53T α-synuclein (hA53T) transgenic mouse model of PD (Yun et al., 2018) It was shown that the GLP-1R agonist, NLY01, protects against dopaminergic neuronal loss and behavioral deficits in a mouse model of sporadic PD and extends survival in a model of familial α-synucleinopathy. NLY01 prevented microglial secretion of IL-1α, TNFα, and C1q, decreased expression of several A1 astrocyte genes (*H2-T23, Serping1, H2-D1, Ggta1, Iigp1, Gbp2, Fbln5, Ugt1a, Fkbp5, Psmb8, Srgn, and Amigo2*) and attenuated microglia activation and A1 astrogliosis evidenced by reduced number of Iba-1^+^ and C3d^+^GFAP^+^ cells (Yun et al., 2018). These results clearly show that the protective effects of NLY01 were attributed to the inhibition of microglial activation, and subsequent attenuation of cytotoxic reactive astrogliosis, a process implicated in neurodegeneration. NLY01, designed to reduce inflammation by targeting microglia and astrocytes, showed promise in slowing disease progression and extending lifespan in animal models, and was well-tolerated in a Phase I trial (NCT03672604) for healthy adults. However, in a Phase II clinical trial known as NLY01-PD-1 (NCT04154072) for early and untreated PD, it did not demonstrate superiority over a placebo in slowing motor symptom progression overall. Nevertheless, there is intrigue as NLY01 showed a beneficial effect in patients under 60, suggesting potential interest for further clinical evaluation in younger individuals with PD and other dementing conditions.

In transgenic mouse models of AD, 5xFAD and 3xTg-AD, subcutaneous injection of NLY01 (1 or 10 mg/kg; twice a week) for 4 months significantly reduced cognitive deficits confirmed by MWM (Park et al., 2021). NLY01 reduced pathologic oligomeric Aβ_1-42_-induced microglia activation and secretion of IL-1α, TNFα, and C1q, subsequently reduced reactive astrogliosis (Park et al., 2021). Researchers involved in advancing NLY01, has commenced a Phase IIB Alzheimer’s study, characterized as a multicenter, randomized, double-blinded, placebo-controlled trial projected to enroll over 500 patients across 100 sites in the US, Canada, and Europe. Additionally, it is in the developmental stage for stroke treatment.

In a mouse model of ischemic stroke, Semaglutide (an FDA approved GLP-1R agonist for type-2 diabetes mellitus) administration (30 nM/kg every 5 days until 28 days) significantly reduced levels of inflammatory cytokines (IL-1α, TNFα, and C1q), Iba-1^+^ microglia/macrophages, and C3d^+^/GFAP^+^ A1 reactive astrocytes (Zhang et al., 2022). Semaglutide-mediated attenuation of reactive astrogliosis resulted in reduced BBB damage, brain infarction and improved neurological function (Zhang et al., 2022). Encouragingly, the GLP-1R agonist Semaglutide is currently in two phase III clinical trials to evaluate the efficacy and safety of Semaglutide in the treatment of dementia (EVOKE, NCT04777396 and EVOKE Plus, NCT04777409). It is also currently in phase 3 trials, aiming to assess its impact on heart disease and stroke in individuals who are overweight or obese (SELECT NCT03574597), which has great significance in dementia considering the role of obesity in contributing to VCID (Buie et al., 2019; Zlokovic et al., 2020).

Therefore, repurposing of FDA-approved GLP-1R agonists with improved pharmacokinetics, could be potential therapeutic strategy for the treatment of neurodegenerative diseases associated with microgliosis and reactive astrogliosis, including AD, PD, PSD, and VCID.

### 4.3 Inflammasome inhibitor

Acuate or chronic cerebral hypoperfusion (CCH)-stimulated inflammasome activation causes reactive astrogliosis and white matter lesions, a major pathology of vascular cognitive impairments (VCI) and dementia (VCID).

In a mouse model of VCID, CCH activates NOD-like receptor family, pyrin domain containing 3 (NLRP3), absent in melanoma 2 (AIM2) inflammasome in reactive astrocytes in white matter tracts in mouse brain, as shown by higher numbers of AIM2 and NLRP3 in GFAP^+^ cells at 4 weeks after BCAS procedure (Matsuyama et al., 2020; Xu et al., 2023). CCH disrupts ionic homeostasis (decreased K+, increased Ca2+, and Na+), increases mitochondrial ROS production and activates NLRP3 inflammasome in VCID (Poh et al., 2022). NLRP3 activation facilitates cleavage of pro-caspase-1 into its active form, leading to the subsequent processing of pro-inflammatory cytokines, notably interleukin-1 beta (IL-1β) and IL-18 within microglia and astrocytes, contributing to the pathogenesis of VCID including white matter lesions (Poh et al., 2021; Li L et al., 2022; Wang et al., 2023). NF-κB signaling activation also induces NLRP3 inflammasome-mediated IL-1β and IL-18 maturation which activate downstream inflammatory cascades including transformation of neurotoxic A1 astrocytes and various cell death pathways including apoptosis, and pyroptosis (Aachoui et al., 2013; Li L et al., 2022; Wang et al., 2023; Wong et al., 2023). In depression-like mice, after 2–6 weeks of chronic mild stress (CMS), the NLRP3 inflammasome activates microglia, leading to the release of an A1 cocktail l (TNF-α, IL-1α, and C1qA) and upregulation of related A1 astrocyte genes (*H2-T23, Serping1, H2-D1, Ugt1a5, Fkbp5, Ligp1, Fbln5, Ggta1, C3, Gbp2, Pmsb8 and Amigo2*) (Li S et al., 2022). The study demonstrates that NLRP3 inflammasome activation induces neurotoxic A1-like astrocytes, and the caspase-1 pathway plays a crucial role in this process. Additionally, the NF-kB pathway is implicated in the activation of microglial NLRP3 inflammasome and A1-like astrocytes in the depression model. CMS-induced reactive astrogliosis and depression and anxiety-like behavior were attenuated in Nlrp3 Global knockout (Nlrp3^−/−^) mice (Li W et al., 2022). Nlrp3 ablation in Nlrp3^Micro−/−^ and Nlrp3^Astro−/−^ mice shows that NLRP3 inflammasome regulates microglial activation, release of cytokines (TNF-α, IL-1α, and C1q) which promotes transformation of neurotoxic A1 astrocytes, neuronal death, and cognitive impairments in mice (Li S et al., 2022). NLRP3 inflammasome inhibition reduces reactive astrogliosis, a key hallmark of neurodegenerative diseases (Freeman and Ting, 2016; Xu et al., 2023). Pharmacological inhibition of NLRP3 inflammasome have shown therapeutic potential against AD, PD, and VCID (Liang et al., 2022; Xu et al., 2023).

In a mouse model of experimental autoimmune encephalomyelitis (EAE), a selective NLRP3 inflammasome inhibitor, MCC950 significantly reduces astrogliosis in the cortex, corpus callosum, hippocampus, and white matter tract of mouse brain (Hou et al., 2023). MCC950 treatment prevented EAE-induced demyelination as shown by increased NG2+, Oligo2+ cells, and MBP levels and improved cognitive function assessed by MWM test. MCC950 treatment prevented transformation of cytotoxic A1 astrocytes (with reduced C3d expression) whereas enhanced transformation of protective A2 astrocytes (as shown by increased S100A10 expression) in GFAP + astrocytes. Notably, NLRP3 inflammasome inhibitor, MCC950 blocks microglial conversion to M1 microglia, and in turn prevents conversion of cytotoxic A1 reactive astrocytes by activated microglia (Hou et al., 2020; Hou et al., 2023). In transgenic AD mouse model of APP/PS1/Nlrp3^−/−^, NLRP3 deficiency reduces amyloid-β deposition and cognitive dysfunction by microglial mediated amyloid-β phagocytosis (Heneka et al., 2013). In CRND8 APP transgenic mice (TgCRND8) model of AD, NLRP3 inhibitor JC-124 administration (50 mg/kg/day, five times per week for four consecutive weeks) significantly reduces number of Iba-1+ microglia/macrophages and Aβ deposition, although, it does not alter the number of GFAP + astrocytes in the brain (Yin et al., 2018). In traumatic brain injury (TBI) rat model, JC-124 treatment decreases brain inflammation as shown by reduced expression of IL-1β, TNF-alpha, and iNOs (Kuwar et al., 2019). Oral administration of OLT1177, an NLRP3 inhibitor reduces microglial activation and improves cognitive deficits in APP/PS1 mice (Lonnemann et al., 2020).

The leading direct NLRP3 inflammasome inhibitor, RRx-001, is a parenterally administered small molecule with the capability to penetrate the blood-brain barrier (Oronsky et al., 2023). RRx-001 is currently being investigated not only for its anti-inflammatory properties but also as a potential treatment for neurodegenerative diseases such as PD and AD (Jayabalan et al., 2023). Additionally, this promising compound, also known as Nibrozetone, is in the early stages of phase I trials for its use as an experimental radiation sensitizer in individuals with brain metastases as it shields normal cells from harm (BRAINSTORM NCT02215512). Exploring the therapeutic implications of RRx-001 in modifying astrogliosis represents a novel avenue worth investigating for VCID, PSD and AD.

Minocycline (a semisynthetic tetracycline antibiotic) inhibited NLRP3 inflammasome activation, reduced the number of Iba-1+ microglial cells, IL-1β expression, and improves neurological outcome in a rat model of early brain injury (EBI) (Li et al., 2016). In a mouse model of ischemic stroke, minocycline treatment (1–50 mg/kg/day of minocycline for 3–14 consecutive days) inhibits NLRP3 expression in microglia confirmed by reduced number of NLRP3^+^ IBA-1^+^ cells. Minocycline also reduces IL-1β and IL-18 cytokines level and cerebral infract volume (Hayakawa et al., 2008; Lu et al., 2016). A phase II clinical trial is underway to investigate the effectiveness of Minocycline in enhancing neurological outcomes in patients undergoing endovascular revascularization for acute ischemic stroke (NCT05367362) with one of the end points being assessment of instrumental activities of daily living. The rationale behind this study is based on the promising anti-inflammatory and protease inhibitory properties demonstrated by Minocycline in various preclinical stroke models (Sheng et al., 2018).

Taken together, NLRP3 inhibitors clearly show efficacy in attenuating microglial activation and reactive astrogliosis, representing inflammasome inhibitors as potential drug class for the treatment of cerebrovascular related dementia including AD, PSD, and VCID.

### 4.4 Blocking inflammatory cytokines and Aβ deposition

Pro-inflammatory cytokines secreted by microglia including IL-1α, IL-18, TNFα, and C1q play a crucial role in the conversion of inflammatory A1 astrocytes, Aβ deposition, demyelination, and neurodegeneration (Lee et al., 2021; Gao et al., 2023). Pharmacological inhibition or antibody-mediated neutralization pro-inflammatory cytokines have shown therapeutic potential against AD, PSD, and other categories of VCID.

Proinflammatory cytokines, such as IL-1 family, IL-6, and TNF-α, are elevated in the plasma, brains, and cerebrospinal fluid of patients with AD (Cacabelos et al., 1991; Su et al., 2016). IL-1β cytokine is known to be elevated in AD pathology (Di Bona et al., 2008; Forlenza et al., 2009). An IL-1R blocking antibody (anti-IL-1R) significantly alleviates cognitive deficits, markedly attenuates tau pathology, and inhibits amyloid-β formation by decreasing NF-κB transcriptional activity in AD model (Kitazawa et al., 2011). Inhibition of IL-1 signaling reduces several tau kinases including GSK-3β, and p38–MAPK, and phosphorylated tau levels as well as expression of S100B, a marker of inflammatory A1 astrocyte in the brain of AD mice (Kitazawa et al., 2011). Several FDA approved drugs including Rilonacept (IL-1 blocker), Anakinra (IL-1R antagonist) and Canakinumab (IL-1β neutralizing antibody) have been used against rheumatoid arthritis (Arnold et al., 2022). However, their efficacy against neurodegenerative diseases needs to be explored. Interestingly, a Phase II clinical trial, titled “ACTION” (NCT04834388), is investigating the potential of Anakinra to mitigate secondary brain damage following spontaneous hemorrhagic stroke. Moreover, endogenous IL-18 neutralization with anti–IL-18 IgG inhibits cytokine production and NF-κB activation in rat model of vascular injury (Maffia et al., 2006), suggesting its potential for the treatment of dementia of vascular origin.

A C1q-neutralization antibody reduces microglia-dependent synaptic loss and cognitive impairments in a mouse model of LPS-induced neuroinflammation (Wu et al., 2023). Notably, a humanized anti-C1q antibody; ANX005 (immunoglobulin G4 recombinant anti-body) is now in Phase I clinical trials for the treatment of autoimmune and neurodegenerative diseases (Lansita et al., 2017). Endogenous TNFα neutralization also provide neuroprotection against focal ischemic injury (Barone et al., 1997). TNF-α inhibitors, including Etanercept and type-II human TNF-α receptor to a transferrin receptor antibody reduces activation of microglia and tau deposition (Ou et al., 2021). Another TNF-α inhibitors, Adalimumab reduces AD pathology and neurotoxicity by inhibition of NF-κB and improves cognitive deficits (Park et al., 2019). Furthermore, in a rat model of VCID, administration of Adalimumab not only suppressed the activity of NF-κB, but also decreased microglial activation (Xu J et al., 2021). This led to a mitigation of neuronal loss in the hippocampi and an improvement of learning and memory function, providing more evidence supporting the potential of adalimumab for VCID and related dementias.

Recently, FDA approved two anti-amyloid anti-bodies; Aducanumab (human monoclonal antibody) and Lecanemab (humanized IgG1 antibody), which markedly reduce Aβ deposition in brain (Fedele, 2023). Aducanumab neutralizes oligomeric forms of Aβ in cerebral amyloid angiopathy (CAA) lesions. Aducanumab prominently binds with parenchymal Aβ but not vascular Aβ in either cortex or hippocampus and clears Aβ deposits from brains by recruiting Iba1-positive microglia and GFAP-positive astrocyte to Aβ plaques. Microglia and astrocytes clear pathological Aβ deposits through phagocytosis and degrades in CNS and moderately improves spatial working memory (Ries and Sastre, 2016; Sevigny et al., 2016; Kong et al., 2022). Lecanemab, an anti-Aβ protofibril antibody, demonstrates efficacy in reducing cognitive decline in early AD patients; however, its mechanism for Aβ plaque clearance remains inadequately understood (Prillaman, 2022; van Dyck et al., 2023). Lecanemab is the subject of multiple clinical trials including phase III studies for early-stage AD and disease progression (NCT03887455, NCT05925621, NCT04468659, NCT01767311, NCT05269394, etc.). Gantenerumab, a subcutaneously administered anti-Aβ IgG1 monoclonal antibody with high affinity for aggregated Aβ, demonstrated promising results in reducing amyloid plaque burden in early AD; however, it did not show a significant impact on slowing clinical decline compared to a placebo at 116 weeks (Bateman et al., 2023). Solanezumab, designed to target monomeric amyloid in individuals with elevated brain amyloid levels, did not demonstrate a reduction in cognitive decline compared to a placebo over a 240-week period in those with preclinical AD (Sperling et al., 2023). The drug’s mechanism involves binding to the central epitope of monomeric amyloid-β, known as the nucleation site for Aβ oligomerization. Acting as an “amyloid beta sink,” Solanezumab facilitates the flux of amyloid beta from a central to peripheral compartment, increasing peripheral elimination and solubilizing amyloid-β in the cerebrospinal fluid to reestablish equilibrium (Willis et al., 2018). The potential efficacy of these drugs in the broader context of astrogliosis and VCID including PSD remains unexplored.

These studies illustrate the potential therapeutic efficacy of antibodies neutralizing pro-inflammatory cytokines, receptors associated with inflammation, and anti-amyloid antibodies in addressing cerebrovascular and neurodegenerative diseases, including AD, PSD, and various forms of VCID.

### 4.5 Choline-containing phospholipids

Choline-containing phospholipids, influencing transglutaminase activity, may impact astrocyte differentiation, proliferation, and reactivity (Bramanti et al., 2008; Tayebati et al., 2009). Understanding these astrocyte functions offers valuable insights into neurodegenerative processes and potential therapeutic interventions (Lee et al., 2022; Lo et al., 2022). Choline alphoscerate (alpha glyceryl phosphorylcholine, α-GPC), a cognition-enhancing choline-containing phospholipid, is explored for countering cognitive impairment in conditions like AD and stroke (Tayebati, Di Tullio, Tomassoni and Amenta, 2009). Recognized as a parasympathetic agent, α-GPC, used as a registered drug or nutraceutical (Parker et al., 2022), has demonstrated in preclinical studies to increase acetylcholine release (Wang et al., 2009), improve learning and memory, and is under evaluation in clinical trials for efficacy and Safety in VCID Patients (NCT05050604) (Salvadori et al., 2021; Jeon et al., 2023). Choline-containing phospholipids emerge as an unexplored category of drugs in the context of PSD and reactive astrogliosis.

## 5 Therapeutic challenges and future directions

The development of novel treatments for cognitive health conditions, including PSD, AD, and other VCIDs, faces numerous obstacles. These include challenges associated with multiple comorbidities, various neurodegenerative pathologies, blood-brain barrier (BBB) permeability, polypharmacy, and disease specificity.

Neuronal and glial cation-chloride cotransporters, particularly NKCC1, are promising targets for central nervous system (CNS) drugs (Löscher and Kaila, 2022). However, significant challenges exist in drug design, including isoform specificity, pharmacokinetics, and safety properties. Bumetanide, often considered an NKCC1 blocker, lacks specificity between NKCC1 and NKCC2. Off-target effect is also another concern. For example, in the case of Bumetanide, the induction of diuresis, concomitant risk for hypokalemic alkalosis and potential ototoxicity clearly limits the application of bumetanide for chronic treatment of CNS disorders (Sica, 2004; Auer et al., 2020).

One significant challenge in designing CNS drugs is their ability to effectively cross the BBB (Banks, 2008). Existing NKCC1 inhibitors face poor blood-brain barrier permeability (Löscher and Kaila, 2022). For example, bumetanide encounters pharmacokinetic constraints due to its limited BBB penetration. Strategies like using lipophilic prodrugs or derivatives aim to enhance central nervous system (CNS) delivery (Auer, Schreppel, Erker and Schwarzer, 2020) but may introduce associated toxicity risks, as seen with STS5/BUM5 in stroke models (Huang et al., 2019).

In certain conditions like stroke, ischemia-induced BBB leakage presents an opportunity for drug delivery, albeit with unpredictable results (Ronaldson et al., 2024). Changes in BBB permeability can lead to ionic imbalances and the accumulation of toxic metabolic substances, disrupting synaptic, neuronal, and oligodendrocyte functions. Moreover, impaired glucose transport across the BBB and reduced regional metabolic rates in AD (Patching, 2017) further complicate treatment approaches.

The complexity of dementia, compounded by multiple comorbidities and pathologies, impedes the efficacy of single-target treatments (Park et al., 2017; Growdon et al., 2021; Riedl et al., 2022). Polypharmacy in patients exacerbates treatment challenges, increasing the risk of adverse reactions (Hohl et al., 2001; Doumat et al., 2023). Clinical trial recruitment often excludes individuals with multiple conditions, despite representing a significant real-world cohort, potentially leading to disparities between trial outcomes and real-world application. Additionally, disease-specific drugs may limit their applicability across different dementia types. For instance, cholinesterase inhibitors show limited efficacy in VCID (Battle et al., 2021) compared to AD (Xu H et al., 2021; Majidazar et al., 2022), and some drugs like memantine is only recommended for Alzheimer’s (Levine and Langa, 2011).

Despite recent successes in immunotherapy trials, challenges persist, including patient non-response and disease recurrence due to evolving resistance. Age-related immune system impairment may further complicate immunotherapy outcomes in elderly patients (Castelo-Branco and Soveral, 2014; Weyand and Goronzy, 2016).

Amyloid-related imaging abnormalities (ARIA) represents a major side effect of AD immunotherapy (Sperling et al., 2011; Withington and Turner, 2022). Identifying early biomarkers of ARIA represent an important challenge to ensure safe and beneficial effects of immunotherapies, given that different promising clinical trials in prodromal and subjects at risk for AD are underway (DiFrancesco et al., 2015). ARIA is a term introduced in 2010 to encompass a spectrum of MRI findings observed in patients receiving investigational anti–amyloid beta (Aβ) immunotherapies for AD. The two types of amyloid-related imaging abnormalities (ARIA) present distinct characteristics: ARIA-E, identified by magnetic resonance imaging (MRI) showing evidence of vasogenic edema (VE) and/or sulcal effusion on fluid-attenuated inversion recovery (FLAIR), indicating inflammation at the affected vessels; and ARIA-H, distinguished by hemosiderin deposits, microhemorrhages (MHs), and superficial siderosis on T2*-weighted gradient echo (T2*-GRE) or susceptibility-weighted imaging (SWI), indicative of cerebral amyloid angiopathy (CAA) (Barakos et al., 2013). The ARIA concern has garnered heightened attention following the presentation of highly promising Phase 1b trial data for aducanumab (NCT01677572) (Salloway et al., 2022). The recent revelation that cerebrospinal fluid (CSF) anti-Aβ autoantibodies contribute significantly to the development of ARIA-like events associated with cerebral amyloid angiopathy-related inflammation has sparked considerable interest in the realm of immunotherapy (DiFrancesco, Longoni and Piazza, 2015).

In conclusion, tackling these challenges necessitates a comprehensive approach that takes into account the varied needs and intricacies of patients with cognitive impairments, and entails tailored designs for both preclinical and clinical studies.

## 6 Concluding remarks

In summary, this review highlights the intricate involvement of astrocytes in cerebrovascular diseases and neurodegenerative disorders, specifically focusing on VCIDs such as PSD and also AD. The interplay between A1 and A2 astrocytes in reactive astrogliosis, notably the NKCC1-related ion transporter cascade, emerges as a promising therapeutic target. The review explores strategies, including inhibitors and agonists of signaling molecules, to address cognitive impairments. Despite these positive developments, the critical need for effective therapies for dementing disorders is emphasized, given the substantial burden on healthcare systems in an aging world. Ongoing research is crucial to formulate targeted interventions such as the SPAK inhibitor ZT-1a, capable of modulating astrocyte signaling and mitigating factors contributing to neurodegenerative diseases leading to dementia (VCID and AD). A multifaceted approach may hold the key to advancing strategies against these formidable yet partially reversible disorders.

## Figures and Tables

**Table:** 

**AD**	Alzheimer’s disease
**APOE4**	Apolipoprotein E4
**APP**	Amyloid precursor protein
**ATP**	Adenosine triphosphate
**Aβ**	Amyloid beta
**BBB**	Blood–brain barrier
**BCAS**	Bilateral common carotid artery stenosis
**C1q**	complement component 1q
**C3**	complement component 3
**CAA**	Cerebral amyloid angiopathy
**CCH**	Chronic cerebral hypoperfusion
**CMS**	Chromic mild stress
**CNS**	Central nervous system
**GFAP**	Glial fibrillary acidic protein
**GLP**	Glucagon-like peptide
**GLUT1**	Glucose transporter type 1
**hA53T**	human A53T α-synuclein
**Iba1**	Ionized calcium binding adaptor molecule 1
**IL**	interleukin
**LCN2**	Lipocalin 2
**LPS**	lipopolysaccharides
**MBP**	Myelin basic protein
**MRI**	Magnetic resonance imaging
**MWM**	Morris water maze
**NF-kB**	Nuclear factor kappa B
**NG2**	Neural/glial antigen 2
**NHE1**	Na+/H+ exchanger isoform 1
**NKCC1**	Na + -K + -2Cl-cotransporter 1
**NLRP3**	NOD-like receptor protein 3
**OL**	Oligodendrocytes
**Oligo2**	oligodendrocyte transcription factor 2
**OPC**	Oligodendrocyte progenitor cell
**OPCs**	Oligodendrocyte precursor cells
**OSR1**	Oxidative stress-responsive kinase 1
**PD**	Parkinson’s disease
**PSD**	post-stroke dementia
**ROS**	Reactive oxygen species
**S100A10**	S100 calcium-binding protein A10
**SPAK**	The STE20/SPS1-related proline/alanine-rich kinase
**TBI**	traumatic brain injury
**TGF-β**	Transforming growth factor β
**TNFα**	Tumor necrosis factor alpha
**VCID**	Vascular contributions to cognitive impairment and dementia
**WML**	White matter lesions
**WNK**	With no lysine(K) kinase
**α-GPC**	Alpha glyceryl phosphorylcholine

## References

[B1] Aachoui Y. Sagulenko V. Miao E. A. Stacey K. J. (2013). Inflammasome-mediated pyrophoric and apoptotic cell death, and defense against infection. Curr. Opin. Microbiol. 16 (3), 319–326. 10.1016/j.mib.2013.04.004 23707339 PMC3742712

[B2] Allen N. J. Eroglu C. (2017). Cell biology of astrocyte-synapse interactions. Neuron 96 (3), 697–708. 10.1016/j.neuron.2017.09.056 29096081 PMC5687890

[B3] Alzheimer A. (1910). Die diagnostischen schwierigkeiten in der Psychiatric. Z. für gesamte Neurol. Psychiatr. 1 (1), 1–19. 10.1007/bf02895916

[B4] Annunziato L. Boscia F. Pignataro G. (2013). Ionic transporter activity in astrocytes, microglia, and oligodendrocytes during brain ischemia. J. Cereb. Blood Flow. Metab. 33 (7), 969–982. 10.1038/jcbfm.2013.44 23549380 PMC3705429

[B5] Arnold D. D. Yalamanoglu A. Boyman O. (2022). Systematic review of safety and efficacy of IL-1-targeted Biologics in treating immune-mediated disorders. Front. Immunol. 13, 888392. 10.3389/fimmu.2022.888392 35874710 PMC9296857

[B6] Arranz A. M. De Strooper B. (2019). The role of astroglia in Alzheimer's disease: pathophysiology and clinical implications. Lancet Neurol. 18 (4), 406–414. 10.1016/S1474-4422(18)30490-3 30795987

[B7] Athauda D. Maclagan K. Skene S. S. Bajwa-Joseph M. Letchford D. Chowdhury K. (2017). Eventide once weekly versus placebo in Parkinson's disease: a randomised, double-blind, placebo-controlled trial. Lancet 390 (10103), 1664–1675. 10.1016/S0140-6736(17)31585-4 28781108 PMC5831666

[B8] Auer T. Schreppel P. Erker T. Schwarzer C. (2020). Functional characterization of novel bumetanide derivatives for epilepsy treatment. Neuropharmacology 162, 107754. 10.1016/j.neuropharm.2019.107754 31476353

[B9] Aviles-Olmos I. Dickson J. Kefalopoulou Z. Djamshidian A. Ell P. Soderlund T. (2013). Eventide and the treatment of patients with Parkinson's disease. J. Clin. Invest. 123 (6), 2730–2736. 10.1172/JCI68295 23728174 PMC3668846

[B10] Banks W. A. (2008). Developing drugs that can cross the blood-brain barrier: applications to Alzheimer's disease. BMC Neurosci. 9 (3), S2. 10.1186/1471-2202-9-s3-s2 PMC2604887 19090999

[B11] Barakos J. Sperling R. Salloway S. Jack C. Gass A. Fiebach J. B. (2013). MR imaging features of amyloid-related imaging abnormalities. AJNR Am. J. Neuroradiol. 34 (10), 1958–1965. 10.3174/ajnr.A3500 23578674 PMC7965435

[B12] Barone F. C. Arvin B. White R. F. Miller A. Webb C. L. Willette R. N. (1997). Tumor necrosis factor-alpha. A mediator of focal ischemic brain injury. Stroke 28 (6), 1233–1244. 10.1161/01.str.28.6.1233 9183357

[B13] Bateman R. J. Smith J. Donohue M. C. Delmar P. Abbas R. the Gantenerumab Study G. (2023). Two phase 3 trials of Gantenerumab in early Alzheimer's disease. N. Engl. J. Med. 389 (20), 1862–1876. 10.1056/NEJMoa2304430 37966285 PMC10794000

[B14] Battle C. E. Abdul-Rahim A. H. Shenkin S. D. Hewitt J. Quinn T. J. (2021). Cholinesterase inhibitors for vascular dementia and other vascular cognitive impairments: a network meta-analysis. Cochrane Database Syst. Rev. 2 (2), Cd013306. 10.1002/14651858.CD013306.pub2 33704781 PMC8407366

[B15] Begum G. Song S. Wang S. Zhao H. Bhuiyan M. I. H. Li E. (2018). Selective knockout of astrocytic Na(+)/H(+) exchanger isoform 1 reduces astrogliosis, BBB damage, infarction, and improves neurological function after ischemic stroke. Glia 66 (1), 126–144. 10.1002/glia.23232 28925083 PMC5705024

[B16] Begum G. Yuan H. Kahle K. T. Li L. L. Wang S. X. Shi Y. J. (2015). Inhibition of WNK3 kinase signaling reduces brain damage and accelerates neurological recovery after stroke. Stroke 46 (7), 1956–1965. <Go to ISI>://WOS:000356672800030. 10.1161/STROKEAHA.115.008939 26069258 PMC4643659

[B17] Bélanger M. Allaman I. Magistretti P. J. (2011). Brain energy metabolism: focus on astrocyte-neuron metabolic cooperation. Cell Metab. 14 (6), 724–738. 10.1016/j.cmet.2011.08.016 22152301

[B18] Belanger M. Magistretti P. J. (2009). The role of astroglia in neuroprotection. Dialogues Clin. Neurosci. 11 (3), 281–295. 10.31887/DCNS.2009.11.3/mbelanger 19877496 PMC3181926

[B19] Bhuiyan M. I. H. Fischer S. Patel S. M. Oft H. Zhang T. Foley L. M. (2023). Efficacy of novel SPAK inhibitor ZT-1a derivatives (1c, 1d, 1g and 1h) on improving post-stroke neurological outcome and brain lesion in mice. Neurochem. Int. 162, 105441. 10.1016/j.neuint.2022.105441 36375633 PMC9839627

[B20] Bhuiyan M. I. H. Habib K. Sultan M. T. Chen F. Jahan I. Weng Z. (2024a). SPAK inhibitor ZT-1a attenuates reactive astrogliosis and oligodendrocyte degeneration in a mouse model of vascular dementia. CNS Neurosci. Ther. 30 (3), e14654. 10.1111/cns.14654 38433018 PMC10909630

[B21] Bhuiyan M. I. H. Khadija H. Sultan M. Chen F. Weng Z. Rahman M. (2024b). “SPAK inhibitor ZT-1a attenuates reactive astrogliosis and oligodendrocyte degeneration in a mouse model of vascular dementia,” in In revision. 10.1111/cns.14654 PMC10909630 38433018

[B22] Bhuiyan M. I. H. Song S. Yuan H. Begum G. Kofler J. Kahle K. T. (2017). WNK-Cab39-NKCC1 signaling increases the susceptibility to ischemic brain damage in hypertensive rats. J. Cereb. Blood Flow. Metab. 37 (8), 2780–2794. 10.1177/0271678X16675368 27798271 PMC5536788

[B23] Bhuiyan M. I. H. Young C. B. Jahan I. Hasan M. N. Fischer S. Meor Azlan N. F. (2022). NF-κB signaling-mediated activation of WNK-SPAK-NKCC1 cascade in worsened stroke outcomes of Ang II-hypertensive mice. Stroke 53 (5), 1720–1734. 10.1161/STROKEAHA.121.038351 35272484 PMC9038703

[B24] Bi F. Huang C. Tong J. Qiu G. Huang B. Wu Q. (2013). Reactive astrocytes secrete lcn2 to promote neuron death. Proc. Natl. Acad. Sci. U. S. A. 110 (10), 4069–4074. 10.1073/pnas.1218497110 23431168 PMC3593910

[B25] Boscia F. Begum G. Pignataro G. Sirabella R. Cuomo O. Casamassa A. (2016). Glial Na(+) -dependent ion transporters in pathophysiological conditions. Glia 64 (10), 1677–1697. 10.1002/glia.23030 27458821 PMC5238576

[B26] Boyarko B. Podvin S. Greenberg B. Momper J. D. Huang Y. Gerwick W. H. (2023). Evaluation of bumetanide as a potential therapeutic agent for Alzheimer's disease. Front. Pharmacol. 14, 1190402. 10.3389/fphar.2023.1190402 37601062 PMC10436590

[B27] Bramanti V. Bronzi D. Tomassoni D. Li Volti G. Cannavo G. Raciti G. (2008). Effect of choline-containing phospholipids on transglutaminase activity in primary astroglial cell cultures. Clin. Exp. Hypertens. 30 (8), 798–807. 10.1080/10641960802563576 19021029

[B28] Brambilla R. Bracchi-Ricard V. Hu W. H. Frydel B. Bramwell A. Karmally S. (2005). Inhibition of astroglial nuclear factor kappaB reduces inflammation and improves functional recovery after spinal cord injury. J. Exp. Med. 202 (1), 145–156. 10.1084/jem.20041918 15998793 PMC2212896

[B29] Brandt C. Seja P. Töllner K. Römermann K. Hampel P. Kalesse M. (2018). Bumepamine, a brain-permeant benzylamine derivative of bumetanide, does not inhibit NKCC1 but is more potent to enhance phenobarbital's anti-seizure efficacy. Neuropharmacology 143, 186–204. 10.1016/j.neuropharm.2018.09.025 30248303

[B30] Brown A. M. Ransom B. R. (2007). Astrocyte glycogen and brain energy metabolism. Glia 55 (12), 1263–1271. 10.1002/glia.20557 17659525

[B31] Buie J. J. Watson L. S. Smith C. J. Sims-Robinson C. (2019). Obesity-related cognitive impairment: the role of endothelial dysfunction. Neurobiol. Dis. 132, 104580. 10.1016/j.nbd.2019.104580 31454547 PMC6834913

[B32] Cacabelos R. Barquero M. Garcia P. Alvarez X. A. Varela de Seijas E. (1991). Cerebrospinal fluid interleukin-1 beta (IL-1 beta) in Alzheimer's disease and neurological disorders. Methods Find. Exp. Clin. Pharmacol. 13 (7), 455–458. https://www.ncbi.nlm.nih.gov/pubmed/1784142. 1784142

[B33] Castelo-Branco C. Soveral I. (2014). The immune system and aging: a review. Gynecol. Endocrinol. 30 (1), 16–22. 10.3109/09513590.2013.852531 24219599

[B34] Cataldi R. Sachdev P. S. Chowdhary N. Seeher K. Bentvelzen A. Moorthy V. (2023). A WHO blueprint for action to reshape dementia research. Nat. Aging 3 (5), 469–471. 10.1038/s43587-023-00381-6 37202512

[B35] Chen A. Akinyemi R. O. Hase Y. Firbank M. J. Ndung'u M. N. Foster V. (2016). Frontal white matter hyperintensities, clasmatodendrosis and gliovascular abnormalities in ageing and post-stroke dementia. Brain 139 (1), 242–258. 10.1093/brain/awv328 26667280 PMC4905522

[B36] Christopherson K. S. Ullian E. M. Stokes C. C. Mullowney C. E. Hell J. W. Agah A. (2005). Thrombospondin are astrocyte-secreted proteins that promote CNS synaptogenesis. Cell 120 (3), 421–433. 10.1016/j.cell.2004.12.020 15707899

[B37] Chun H. Lee C. J. (2018). Reactive astrocytes in Alzheimer's disease: a double-edged sword. Neurosci. Res. 126, 44–52. 10.1016/j.neures.2017.11.012 29225140

[B38] Clarke L. E. Liddelow S. A. Chakraborty C. Munch A. E. Heiman M. Barres B. A. (2018). Normal aging induces A1-like astrocyte reactivity. Proc. Natl. Acad. Sci. U. S. A. 115 (8), E1896–E1905. 10.1073/pnas.1800165115 29437957 PMC5828643

[B39] Corriveau R. A. Bosetti F. Emr M. Gladman J. T. Koenig J. I. Moy C. S. (2016). The science of vascular contributions to cognitive impairment and dementia (VCID): a framework for advancing research priorities in the cerebrovascular biology of cognitive decline. Cell Mol. Neurobiol. 36 (2), 281–288. 10.1007/s10571-016-0334-7 27095366 PMC4859348

[B40] Dalakas M. C. Alexopoulos H. Spaeth P. J. (2020). Complement in neurological disorders and emerging complement-targeted therapeutics. Nat. Rev. Neurol. 16 (11), 601–617. 10.1038/s41582-020-0400-0 33005040 PMC7528717

[B41] Desagher S. Glowinski J. Premont J. (1996). Astrocytes protect neurons from hydrogen peroxide toxicity. J. Neurosci. 16 (8), 2553–2562. 10.1523/JNEUROSCI.16-08-02553.1996 8786431 PMC6578753

[B42] De Strooper B. Karran E. (2016). The cellular phase of Alzheimer's disease. Cell 164 (4), 603–615. 10.1016/j.cell.2015.12.056 26871627

[B43] Di Bona D. Plaia A. Vasto S. Cavallone L. Lescai F. Franceschi C. (2008). Association between the interleukin-1beta polymorphisms and Alzheimer's disease: a systematic review and meta-analysis. Brain Res. Rev. 59 (1), 155–163. 10.1016/j.brainresrev.2008.07.003 18675847

[B44] DiFrancesco J. C. Longoni M. Piazza F. (2015). Anti-aβ autoantibodies in amyloid related imaging abnormalities (ARIA): candidate biomarker for immunotherapy in Alzheimer's disease and cerebral amyloid angiopathy. Front. Neurol. 6, 207. 10.3389/fneur.2015.00207 26441825 PMC4585101

[B45] Diz-Chaves Y. Herrera-Perez S. Gonzalez-Matias L. C. Lamas J. A. Mallo F. (2020). Glucagon-like peptide-1 (GLP-1) in the integration of neural and endocrine responses to stress. Nutrients 12 (11), 3304. 10.3390/nu12113304 33126672 PMC7692797

[B46] Doumat G. Daher D. Itani M. Abdouni L. El Asmar K. Assaf G. (2023). The effect of polypharmacy on healthcare services utilization in older adults with comorbidities: a retrospective cohort study. BMC Prim. Care 24 (1), 120. 10.1186/s12875-023-02070-0 37237338 PMC10214698

[B47] Dringen R. Kussmaul L. Gutterer J. M. Hirrlinger J. Hamprecht B. (1999). The glutathione system of peroxide detoxification is less efficient in neurons than in astroglial cells. J. Neurochem. 72 (6), 2523–2530. 10.1046/j.1471-4159.1999.0722523.x 10349863

[B48] Erker T. Brandt C. Töllner K. Schreppel P. Twele F. Schidlitzki A. (2016). The bumetanide prodrug BUM5, but not bumetanide, potentiates the antiseizure effect of phenobarbital in adult epileptic mice. Epilepsia 57 (5), 698–705. 10.1111/epi.13346 26921222

[B49] Escartin C. Galea E. Lakatos A. O'Callaghan J. P. Petzold G. C. Serrano-Pozo A. (2021). Reactive astrocyte nomenclature, definitions, and future directions. Nat. Neurosci. 24 (3), 312–325. 10.1038/s41593-020-00783-4 33589835 PMC8007081

[B50] Escartin C. Guillemaud O. Carrillo-de Sauvage M. A. (2019). Questions and (some) answers on reactive astrocytes. Glia 67 (12), 2221–2247. 10.1002/glia.23687 31429127

[B51] Fedele E. (2023). Anti-amyloid therapies for Alzheimer's disease and the amyloid cascade hypothesis. Int. J. Mol. Sci. 24 (19), 14499. 10.3390/ijms241914499 37833948 PMC10578107

[B52] Fei X. Dou Y. N. Wang L. Wu X. Huan Y. Wu S. (2022). Homer1 promotes the conversion of A1 astrocytes to A2 astrocytes and improves the recovery of transgenic mice after intracerebral hemorrhage. J. Neuroinflammation 19 (1), 67. 10.1186/s12974-022-02428-8 35287697 PMC8922810

[B53] Forlenza O. V. Diniz B. S. Talib L. L. Mendonca V. A. Ojopi E. B. Gattaz W. F. (2009). Increased serum IL-1beta level in Alzheimer's disease and mild cognitive impairment. Dement. Geriatr. Cogn. Disord. 28 (6), 507–512. 10.1159/000255051 19996595

[B54] Freeman L. C. Ting J. P.-Y. (2016). The pathogenic role of the inflammasome in neurodegenerative diseases. J. Neurochem. 136 (S1), 29–38. 10.1111/jnc.13217 26119245

[B55] Gao C. Jiang J. Tan Y. Chen S. (2023). Microglia in neurodegenerative diseases: mechanism and potential therapeutic targets. Signal Transduct. Target Ther. 8 (1), 359. 10.1038/s41392-023-01588-0 37735487 PMC10514343

[B56] Garcia-Caceres C. Quarta C. Varela L. Gao Y. Gruber T. Legutko B. (2016). Astrocytic insulin signaling couples brain glucose uptake with nutrient availability. Cell 166 (4), 867–880. 10.1016/j.cell.2016.07.028 27518562 PMC8961449

[B57] Gloeckner C. Garner A. L. Mersha F. Oksov Y. Tricoche N. Eubanks L. M. (2010). Repositioning of an existing drug for the neglected tropical disease Onchocerciasis. Proc. Natl. Acad. Sci. U. S. A. 107 (8), 3424–3429. 10.1073/pnas.0915125107 20142509 PMC2840490

[B58] Gonzalez-Garcia I. Gruber T. Garcia-Caceres C. (2021). Insulin action on astrocytes: from energy homeostasis to behaviour. J. Neuroendocrinol. 33 (4), e12953. 10.1111/jne.12953 33724579

[B59] Gorelick P. B. Scuteri A. Black S. E. Decarli C. Greenberg S. M. Iadecola C. (2011). Vascular contributions to cognitive impairment and dementia: a statement for healthcare professionals from the american heart association/american stroke association. Stroke 42 (9), 2672–2713. 10.1161/STR.0b013e3182299496 21778438 PMC3778669

[B60] Gottesman R. F. Albert M. S. Alonso A. Coker L. H. Coresh J. Davis S. M. (2017). Associations between midlife vascular risk factors and 25-year incident dementia in the atherosclerosis risk in communities (ARIC) cohort. JAMA Neurol. 74 (10), 1246–1254. 10.1001/jamaneurol.2017.1658 28783817 PMC5710244

[B61] Graber-Naidich A. Lee J. Younes K. Greicius M. D. Le Guen Y. He Z. (2023a). Bumetanide exposure association with Alzheimer's disease risk. Res. Sq. 10.21203/rs.3.rs-2574215/v1 PMC10563814 37822457

[B62] Graber-Naidich A. Lee J. Younes K. Greicius M. D. Le Guen Y. He Z. (2023b). Loop diuretics association with Alzheimer's disease risk. Front. Aging 4, 1211571. 10.3389/fragi.2023.1211571 37822457 PMC10563814

[B63] Growdon M. E. Gan S. Yaffe K. Steinman M. A. (2021). Polypharmacy among older adults with dementia compared with those without dementia in the United States. J. Am. Geriatr. Soc. 69 (9), 2464–2475. 10.1111/jgs.17291 34101822 PMC8440349

[B64] Guo H. Fan Z. Wang S. Ma L. Wang J. Yu D. (2021). Astrocytic A1/A2 paradigm participates in glycogen mobilization mediated neuroprotection on reperfusion injury after ischemic stroke. J. Neuroinflammation 18 (1), 230. 10.1186/s12974-021-02284-y 34645472 PMC8513339

[B65] Habib N. McCabe C. Medina S. Varshavsky M. Kitsberg D. Dvir-Szternfeld R. (2020). Disease-associated astrocytes in Alzheimer's disease and aging. Nat. Neurosci. 23 (6), 701–706. 10.1038/s41593-020-0624-8 32341542 PMC9262034

[B66] Hainsworth A. H. Elahi F. M. Corriveau R. A. (2021). An introduction to therapeutic approaches to vascular cognitive impairment. Cereb. Circ. Cogn. Behav. 2, 100033. 10.1016/j.cccb.2021.100033 34950896 PMC8661126

[B67] Hasel P. Rose I. V. L. Sadick J. S. Kim R. D. Liddelow S. A. (2021). Neuroinflammatory astrocyte subtypes in the mouse brain. Nat. Neurosci. 24 (10), 1475–1487. 10.1038/s41593-021-00905-6 34413515

[B68] Hayakawa K. Mishima K. Nozako M. Hazekawa M. Mishima S. Fujioka M. (2008). Delayed treatment with minocycline ameliorates neurologic impairment through activated microglia expressing a high-mobility group box1-inhibiting mechanism. Stroke 39 (3), 951–958. 10.1161/STROKEAHA.107.495820 18258837

[B69] Heneka M. T. Kummer M. P. Stutz A. Delekate A. Schwartz S. Vieira-Saecker A. (2013). NLRP3 is activated in Alzheimer's disease and contributes to pathology in APP/PS1 mice. Nature 493 (7434), 674–678. 10.1038/nature11729 23254930 PMC3812809

[B70] Hohl C. M. Dankoff J. Colacone A. Afilalo M. (2001). Polypharmacy, adverse drug-related events, and potential adverse drug interactions in elderly patients presenting to an emergency department. Ann. Emerg. Med. 38 (6), 666–671. 10.1067/mem.2001.119456 11719747

[B71] Hosoki S. Hansra G. K. Jayasena T. Poljak A. Mather K. A. Catts V. S. (2023). Molecular biomarkers for vascular cognitive impairment and dementia. Nat. Rev. Neurol. 19 (12), 737–753. 10.1038/s41582-023-00884-1 37957261

[B72] Hou B. Yin J. Liu S. Guo J. Zhang B. Zhang Z. (2023). Inhibiting the NLRP3 inflammasome with MCC950 alleviates neurological impairment in the brain of EAE mice. Mol. Neurobiol. 61, 1318–1330. 10.1007/s12035-023-03618-y 37702910 PMC10896958

[B73] Hou B. Zhang Y. Liang P. He Y. Peng B. Liu W. (2020). Inhibition of the NLRP3-inflammasome prevents cognitive deficits in experimental autoimmune encephalomyelitis mice via the alteration of astrocyte phenotype. Cell Death Dis. 11 (5), 377. 10.1038/s41419-020-2565-2 32415059 PMC7229224

[B74] Huang H. Bhuiyan M. I. H. Jiang T. Song S. Shankar S. Taheri T. (2019). A novel Na(+)-K(+)-Cl(-) cotransporter 1 inhibitor STS66* reduces brain damage in mice after ischemic stroke. Stroke 50 (4), 1021–1025. 10.1161/STROKEAHA.118.024287 30862257 PMC6608592

[B75] Huang K. L. Hsiao I. T. Ho M. Y. Hsu J. L. Chang Y. J. Chang T. Y. (2020). Investigation of reactive astrogliosis effect on post-stroke cognitive impairment. J. Neuroinflammation 17 (1), 308. 10.1186/s12974-020-01985-0 33069238 PMC7568828

[B76] Hulshof L. A. van Nuijs D. Hol E. M. Middeldorp J. (2022). The role of astrocytes in synapse loss in Alzheimer's disease: a systematic review. Front. Cell Neurosci. 16, 899251. 10.3389/fncel.2022.899251 35783099 PMC9244621

[B77] Hyvarinen T. Hagman S. Ristola M. Sukki L. Veijula K. Kreutzer J. (2019). Co-stimulation with IL-1β and TNF-α induces an inflammatory reactive astrocyte phenotype with neurosupportive characteristics in a human pluripotent stem cell model system. Sci. Rep. 9 (1), 16944. 10.1038/s41598-019-53414-9 31729450 PMC6858358

[B78] Iadecola C. (2013). The pathobiology of vascular dementia. Neuron 80 (4), 844–866. 10.1016/j.neuron.2013.10.008 24267647 PMC3842016

[B79] Iadecola C. Duering M. Hachinski V. Joutel A. Pendlebury S. T. Schneider J. A. (2019). Vascular cognitive impairment and dementia: JACC scientific expert panel. J. Am. Coll. Cardiol. 73 (25), 3326–3344. 10.1016/j.jacc.2019.04.034 31248555 PMC6719789

[B80] Iadecola C. Yaffe K. Biller J. Bratzke L. C. Faraci F. M. Gorelick P. B. (2016). Impact of hypertension on cognitive function: a scientific statement from the American heart association. Hypertension 68 (6), e67–e94. 10.1161/HYP.0000000000000053 27977393 PMC5361411

[B81] Jayabalan N. Oronsky B. Cabrales P. Reid T. Caroen S. Johnson A. M. (2023). A review of RRx-001: a late-stage multi-indication inhibitor of NLRP3 activation and chronic inflammation. Drugs 83 (5), 389–402. 10.1007/s40265-023-01838-z 36920652 PMC10015535

[B82] Jeon J.-W. Lee S.-Y. Lee S. Han C.-W. Park G. Kim S.-J. (2023). Efficacy and safety of choline alphoscerate for amnestic mild cognitive impairment: a randomized double-blind placebo-controlled trial. 10.21203/rs.3.rs-3488308/v1 PMC11412009 39300341

[B83] Jiwaji Z. Tiwari S. S. Avilés-Reyes R. X. Hooley M. Hampton D. Torvell M. (2022). Reactive astrocytes acquire neuroprotective as well as deleterious signatures in response to Tau and Aß pathology. Nat. Commun. 13 (1), 135. 10.1038/s41467-021-27702-w 35013236 PMC8748982

[B84] Johne M. Käufer C. Römermann K. Gailus B. Gericke B. Löscher W. (2021). A combination of phenobarbital and the bumetanide derivative bumepamine prevents neonatal seizures and subsequent hippocampal neurodegeneration in a rat model of birth asphyxia. Epilepsia 62 (6), 1460–1471. 10.1111/epi.16912 33955541

[B85] Jokinen H. Melkas S. Ylikoski R. Pohjasvaara T. Kaste M. Erkinjuntti T. (2015). Post‐stroke cognitive impairment is common even after successful clinical recovery. Eur. J. neurology 22 (9), 1288–1294. 10.1111/ene.12743 26040251

[B86] Kalaria R. N. Ballard C. (1999). Overlap between pathology of Alzheimer disease and vascular dementia. Alzheimer Dis. Assoc. Disord. 13 (3), S115–S123. 10.1097/00002093-199912003-00017 10609690

[B87] Karimy J. K. Zhang J. Kurland D. B. Theriault B. C. Duran D. Stokum J. A. (2017). Inflammation-dependent cerebrospinal fluid hypersecretion by the choroid plexus epithelium in posthemorrhagic hydrocephalus. Nat. Med. 23 (8), 997–1003. 10.1038/nm.4361 28692063

[B88] Kharod S. C. Kang S. K. Kadam S. D. (2019). Off-label use of bumetanide for brain disorders: an overview. Front. Neurosci. 13, 310. 10.3389/fnins.2019.00310 31068771 PMC6491514

[B89] Kitazawa M. Cheng D. Tsukamoto M. R. Koike M. A. Wes P. D. Vasilevko V. (2011). Blocking IL-1 signaling rescues cognition, attenuates tau pathology, and restores neuronal β-catenin pathway function in an Alzheimer's disease model. J. Immunol. 187 (12), 6539–6549. 10.4049/jimmunol.1100620 22095718 PMC4072218

[B90] Klegeris A. (2021). Targeting neuroprotective functions of astrocytes in neuroimmune diseases. Expert Opin. Ther. Targets 25 (4), 237–241. 10.1080/14728222.2021.1915993 33836642

[B91] Kong C. Yang E. J. Shin J. Park J. Kim S. H. Park S. W. (2022). Enhanced delivery of a low dose of aducanumab via FUS in 5×FAD mice, an AD model. Transl. Neurodegener. 11 (1), 57. 10.1186/s40035-022-00333-x 36575534 PMC9793531

[B92] Kuwar R. Rolfe A. Di L. Xu H. He L. Jiang Y. (2019). A novel small molecular NLRP3 inflammasome inhibitor alleviates neuroinflammatory response following traumatic brain injury. J. Neuroinflammation 16 (1), 81. 10.1186/s12974-019-1471-y 30975164 PMC6458637

[B93] Lansita J. A. Mease K. M. Qiu H. Yednock T. Sankaranarayanan S. Kramer S. (2017). Nonclinical development of ANX005: a humanized anti-C1q antibody for treatment of autoimmune and neurodegenerative diseases. Int. J. Toxicol. 36 (6), 449–462. 10.1177/1091581817740873 29202623

[B94] Lee H. G. Wheeler M. A. Quintana F. J. (2022). Function and therapeutic value of astrocytes in neurological diseases. Nat. Rev. Drug Discov. 21 (5), 339–358. 10.1038/s41573-022-00390-x 35173313 PMC9081171

[B95] Lee J. W. Chun W. Lee H. J. Kim S. M. Min J. H. Kim D. Y. (2021). The role of microglia in the development of neurodegenerative diseases. Biomedicines 9 (10), 1449. 10.3390/biomedicines9101449 34680566 PMC8533549

[B96] Levine D. A. Langa K. M. (2011). Vascular cognitive impairment: disease mechanisms and therapeutic implications. Neurotherapeutics 8 (3), 361–373. 10.1007/s13311-011-0047-z 21556678 PMC3167237

[B97] Leys D. Henon H. Mackowiak-Cordoliani M. A. Pasquier F. (2005). Poststroke dementia. Lancet Neurol. 4 (11), 752–759. 10.1016/S1474-4422(05)70221-0 16239182

[B98] Li J. Chen J. Mo H. Chen J. Qian C. Yan F. (2016). Minocycline protects against NLRP3 inflammasome-induced inflammation and P53-associated apoptosis in early brain injury after subarachnoid hemorrhage. Mol. Neurobiol. 53 (4), 2668–2678. 10.1007/s12035-015-9318-8 26143258

[B99] Liang T. Zhang Y. Wu S. Chen Q. Wang L. (2022). The role of NLRP3 inflammasome in Alzheimer's disease and potential therapeutic targets. Front. Pharmacol. 13, 845185. 10.3389/fphar.2022.845185 35250595 PMC8889079

[B100] Liddelow S. A. Guttenplan K. A. Clarke L. E. Bennett F. C. Bohlen C. J. Schirmer L. (2017). Neurotoxic reactive astrocytes are induced by activated microglia. Nature 541 (7638), 481–487. 10.1038/nature21029 28099414 PMC5404890

[B101] Li K K. Li J. Zheng J. Qin S. (2019). Reactive astrocytes in neurodegenerative diseases. Aging Dis. 10 (3), 664–675. 10.14336/AD.2018.0720 31165009 PMC6538217

[B102] Li L L. Zhou J. Han L. Wu X. Shi Y. Cui W. (2022). The specific role of reactive astrocytes in stroke. Front. Cell Neurosci. 16, 850866. 10.3389/fncel.2022.850866 35321205 PMC8934938

[B103] Li S S. Fang Y. Zhang Y. Song M. Zhang X. Ding X. (2022). Microglial NLRP3 inflammasome activates neurotoxic astrocytes in depression-like mice. Cell Rep. 41 (4), 111532. 10.1016/j.celrep.2022.111532 36288697

[B104] Li T T. Chen X. Zhang C. Zhang Y. Yao W. (2019). An update on reactive astrocytes in chronic pain. J. Neuroinflammation 16 (1), 140. 10.1186/s12974-019-1524-2 31288837 PMC6615111

[B105] Li T T. Liu T. Chen X. Li L. Feng M. Zhang Y. (2020). Microglia induce the transformation of A1/A2 reactive astrocytes via the CXCR7/PI3K/Akt pathway in chronic post-surgical pain. J. Neuroinflammation 17 (1), 211. 10.1186/s12974-020-01891-5 32665021 PMC7362409

[B106] Liu Q. Bhuiyan M. I. H. Liu R. Song S. Begum G. Young C. B. (2021). Attenuating vascular stenosis-induced astrogliosis preserves white matter integrity and cognitive function. J. Neuroinflammation 18 (1), 187. 10.1186/s12974-021-02234-8 34454529 PMC8403348

[B107] Liu Z. Chopp M. (2016). Astrocytes, therapeutic targets for neuroprotection and neurorestoration in ischemic stroke. Prog. Neurobiol. 144, 103–120. 10.1016/j.pneurobio.2015.09.008 26455456 PMC4826643

[B108] Livingston G. Huntley J. Sommerlad A. Ames D. Ballard C. Banerjee S. (2020). Dementia prevention, intervention, and care: 2020 report of the Lancet Commission. Lancet 396 (10248), 413–446. 10.1016/S0140-6736(20)30367-6 32738937 PMC7392084

[B109] Li W. Mandeville E. T. Durán-Laforet V. Fukuda N. Yu Z. Zheng Y. (2022). Endothelial cells regulate astrocyte to neural progenitor cell trans-differentiation in a mouse model of stroke. Nat. Commun. 13 (1), 7812. 10.1038/s41467-022-35498-6 36535938 PMC9763251

[B110] Li X X. Li M. Tian L. Chen J. Liu R. Ning B. (2020). Reactive astrogliosis: implications in spinal cord injury progression and therapy. Oxid. Med. Cell Longev. 2020, 9494352. 10.1155/2020/9494352 32884625 PMC7455824

[B111] Lo J. W. Crawford J. D. Desmond D. W. Bae H. J. Lim J. S. Godefroy O. (2022). Long-term cognitive decline after stroke: an individual participant data meta-analysis. Stroke 53 (4), 1318–1327. 10.1161/STROKEAHA.121.035796 34775838

[B112] Lo J. W. Crawford J. D. Desmond D. W. Godefroy O. Jokinen H. Mahinrad S. (2019). Profile of and risk factors for poststroke cognitive impairment in diverse ethnoregional groups. Neurology 93 (24), e2257–e2271. 10.1212/WNL.0000000000008612 31712368 PMC6937495

[B113] Lo J. W. Crawford J. D. Samaras K. Desmond D. W. Kohler S. Staals J. (2020). Association of prediabetes and type 2 diabetes with cognitive function after stroke: a STROKOG collaboration study. Stroke 51 (6), 1640–1646. 10.1161/STROKEAHA.119.028428 32404039

[B114] Lonnemann N. Hosseini S. Marchetti C. Skouras D. B. Stefanoni D. D'Alessandro A. (2020). The NLRP3 inflammasome inhibitor OLT1177 rescues cognitive impairment in a mouse model of Alzheimer's disease. Proc. Natl. Acad. Sci. U. S. A. 117 (50), 32145–32154. 10.1073/pnas.2009680117 33257576 PMC7749353

[B115] Loomis S. J. Miller R. Castrillo-Viguera C. Umans K. Cheng W. O'Gorman J. (2024). Genome-wide association studies of ARIA from the aducanumab phase 3 ENGAGE and EMERGE studies. Neurology 102 (3), e207919. 10.1212/wnl.0000000000207919 38165296 PMC11097767

[B116] Löscher W. Kaila K. (2022). CNS pharmacology of NKCC1 inhibitors. Neuropharmacology 205, 108910. 10.1016/j.neuropharm.2021.108910 34883135

[B117] Lu Y. Xiao G. Luo W. (2016). Minocycline suppresses NLRP3 inflammasome activation in experimental ischemic stroke. Neuroimmunomodulation 23 (4), 230–238. 10.1159/000452172 27846628

[B118] Luo L. Song S. Ezenwukwa C. C. Jalali S. Sun B. Sun D. (2021). Ion channels and transporters in microglial function in physiology and brain diseases. Neurochem. Int. 142, 104925. 10.1016/j.neuint.2020.104925 33248207 PMC7895445

[B119] Lykke K. Töllner K. Römermann K. Feit P. W. Erker T. MacAulay N. (2015). Structure-activity relationships of bumetanide derivatives: correlation between diuretic activity in dogs and inhibition of the human NKCC2A transporter. Br. J. Pharmacol. 172 (18), 4469–4480. 10.1111/bph.13231 26101812 PMC4562508

[B120] Maffia P. Grassia G. Di Meglio P. Carnuccio R. Berrino L. Garside P. (2006). Neutralization of interleukin-18 inhibits neointimal formation in a rat model of vascular injury. Circulation 114 (5), 430–437. 10.1161/CIRCULATIONAHA.105.602714 16864728

[B121] Magistretti P. J. Allaman I. (2015). A cellular perspective on brain energy metabolism and functional imaging. Neuron 86 (4), 883–901. 10.1016/j.neuron.2015.03.035 25996133

[B122] Mahmoud S. Gharagozloo M. Simard C. Gris D. (2019). Astrocytes maintain glutamate homeostasis in the CNS by controlling the balance between glutamate uptake and release. Cells 8 (2), 184. 10.3390/cells8020184 30791579 PMC6406900

[B123] Majidazar R. Rezazadeh-Gavgani E. Sadigh-Eteghad S. Naseri A. (2022). Pharmacotherapy of Alzheimer's disease: an overview of systematic reviews. Eur. J. Clin. Pharmacol. 78 (10), 1567–1587. 10.1007/s00228-022-03363-6 35881170

[B124] Matsuyama H. Shindo A. Shimada T. Yata K. Wakita H. Takahashi R. (2020). Chronic cerebral hypoperfusion activates AIM2 and NLRP3 inflammasome. Brain Res. 1736, 146779. 10.1016/j.brainres.2020.146779 32171704

[B125] McConnell H. L. Li Z. Woltjer R. L. Mishra A. (2019). Astrocyte dysfunction and neurovascular impairment in neurological disorders: correlation or causation? Neurochem. Int. 128, 70–84. 10.1016/j.neuint.2019.04.005 30986503 PMC7728575

[B126] Metwally S. A. H. Paruchuri S. S. Yu L. Capuk O. Pennock N. Sun D. (2023). Pharmacological inhibition of NHE1 protein increases white matter resilience and neurofunctional recovery after ischemic stroke. Int. J. Mol. Sci. 24 (17), 13289. 10.3390/ijms241713289 37686096 PMC10488118

[B127] Mijajlovic M. D. Pavlovic A. Brainin M. Heiss W. D. Quinn T. J. Ihle-Hansen H. B. (2017). Post-stroke dementia - a comprehensive review. BMC Med. 15 (1), 11. 10.1186/s12916-017-0779-7 28095900 PMC5241961

[B128] Mila-Aloma M. Salvado G. Gispert J. D. Vilor-Tejedor N. Grau-Rivera O. Sala-Vila A. (2020). Amyloid beta, tau, synaptic, neurodegeneration, and glial biomarkers in the preclinical stage of the Alzheimer's continuum. Alzheimers Dement. 16 (10), 1358–1371. 10.1002/alz.12131 32573951 PMC7586814

[B129] Nishida H. Okabe S. (2007). Direct astrocytic contacts regulate local maturation of dendritic spines. J. Neurosci. 27 (2), 331–340. 10.1523/jneurosci.4466-06.2007 17215394 PMC6672072

[B130] Nutma E. van Gent D. Amor S. Peferoen L. A. N. (2020). Astrocyte and oligodendrocyte cross-talk in the central nervous system. Cells 9 (3), 600. 10.3390/cells9030600 32138223 PMC7140446

[B131] O'Donnell M. E. Tran L. Lam T. I. Liu X. B. Anderson S. E. (2004). Bumetanide inhibition of the blood-brain barrier Na-K-Cl cotransporter reduces edema formation in the rat middle cerebral artery occlusion model of stroke. J. Cereb. Blood Flow. Metab. 24 (9), 1046–1056. 10.1097/01.WCB.0000130867.32663.90 15356425

[B132] Oronsky B. Takahashi L. Gordon R. Cabrales P. Caroen S. Reid T. (2023). RRx-001: a chimeric triple action NLRP3 inhibitor, Nrf2 inducer, and nitric oxide superagonist. Front. Oncol. 13, 1204143. 10.3389/fonc.2023.1204143 37313460 PMC10258348

[B133] Ou W. Yang J. Simanauskaite J. Choi M. Castellanos D. M. Chang R. (2021). Biologic TNF-α inhibitors reduce microgliosis, neuronal loss, and tau phosphorylation in a transgenic mouse model of tauopathy. J. Neuroinflammation 18 (1), 312. 10.1186/s12974-021-02332-7 34972522 PMC8719395

[B134] Oveisgharan S. Dawe R. J. Yu L. Kapasi A. Arfanakis K. Hachinski V. (2022). Frequency and underlying pathology of pure vascular cognitive impairment. JAMA Neurol. 79 (12), 1277–1286. 10.1001/jamaneurol.2022.3472 36279115 PMC9593318

[B135] Park H. Y. Park J. W. Song H. J. Sohn H. S. Kwon J. W. (2017). The association between polypharmacy and dementia: a nested case-control study based on a 12-year longitudinal cohort database in South Korea. PLoS One 12 (1), e0169463. 10.1371/journal.pone.0169463 28056068 PMC5215897

[B136] Park J. Lee S. Y. Shon J. Kim K. Lee H. J. Kim K. A. (2019). Adalimumab improves cognitive impairment, exerts neuroprotective effects and attenuates neuroinflammation in an Aβ1-40-injected mouse model of Alzheimer's disease. Cytotherapy 21 (6), 671–682. 10.1016/j.jcyt.2019.04.054 31076196

[B137] Park J. S. Kam T. I. Lee S. Park H. Oh Y. Kwon S. H. (2021). Blocking microglial activation of reactive astrocytes is neuroprotective in models of Alzheimer's disease. Acta Neuropathol. Commun. 9 (1), 78. 10.1186/s40478-021-01180-z 33902708 PMC8074239

[B138] Parker A. G. Byars A. Purpura M. Jäger R. (2022). The effects of alpha-glycerylphosphorylcholine, caffeine or placebo on markers of mood, cognitive function, power, speed, and agility. J. Int. Soc. Sports Nutr. 12 (1), P41. 10.1186/1550-2783-12-s1-p41

[B139] Patching S. G. (2017). Glucose transporters at the blood-brain barrier: function, regulation and gateways for drug delivery. Mol. Neurobiol. 54 (2), 1046–1077. 10.1007/s12035-015-9672-6 26801191

[B140] Patterson C. (2018). World alzheimer report 2018.

[B141] Pelkmans W. Shekari M. Brugulat-Serrat A. Sanchez-Benavides G. Minguillon C. Fauria K. (2024). Astrocyte biomarkers GFAP and YKL-40 mediate early Alzheimer's disease progression. Alzheimers Dement. 20 (1), 483–493. 10.1002/alz.13450 37690071 PMC10917053

[B142] Pendlebury S. T. Rothwell P. M. Oxford Vascular S. (2019). Incidence and prevalence of dementia associated with transient ischaemic attack and stroke: analysis of the population-based Oxford Vascular Study. Lancet Neurol. 18 (3), 248–258. 10.1016/S1474-4422(18)30442-3 30784556 PMC6390174

[B143] Poh L. Fann D. Y. Wong P. Lim H. M. Foo S. L. Kang S. W. (2021). AIM2 inflammasome mediates hallmark neuropathological alterations and cognitive impairment in a mouse model of vascular dementia. Mol. Psychiatry 26 (8), 4544–4560. 10.1038/s41380-020-00971-5 33299135

[B144] Poh L. Sim W. L. Jo D. G. Dinh Q. N. Drummond G. R. Sobey C. G. (2022). The role of inflammasomes in vascular cognitive impairment. Mol. Neurodegener. 17 (1), 4. 10.1186/s13024-021-00506-8 35000611 PMC8744307

[B145] Price B. R. Johnson L. A. Norris C. M. (2021). Reactive astrocytes: the nexus of pathological and clinical hallmarks of Alzheimer's disease. Ageing Res. Rev. 68, 101335. 10.1016/j.arr.2021.101335 33812051 PMC8168445

[B146] Prillaman M. (2022). Alzheimer's drug slows mental decline in trial - but is it a breakthrough? Nature 610 (7930), 15–16. 10.1038/d41586-022-03081-0 36175566

[B147] Rangroo Thrane V. Thrane A. S. Wang F. Cotrina M. L. Smith N. A. Chen M. (2013). Ammonia triggers neuronal disinhibition and seizures by impairing astrocyte potassium buffering. Nat. Med. 19 (12), 1643–1648. 10.1038/nm.3400 24240184 PMC3899396

[B148] Reichenbach N. Delekate A. Plescher M. Schmitt F. Krauss S. Blank N. (2019). Inhibition of Stat3-mediated astrogliosis ameliorates pathology in an Alzheimer's disease model. EMBO Mol. Med. 11 (2), e9665. 10.15252/emmm.201809665 30617153 PMC6365929

[B149] Richardson C. Rafiqi F. H. Karlsson H. K. Moleleki N. Vandewalle A. Campbell D. G. (2008). Activation of the thiazide-sensitive Na+-Cl-cotransporter by the WNK-regulated kinases SPAK and OSR1. J. Cell Sci. 121 (5), 675–684. 10.1242/jcs.025312 18270262

[B150] Riedl L. Kiesel E. Hartmann J. Fischer J. Roßmeier C. Haller B. (2022). A bitter pill to swallow - polypharmacy and psychotropic treatment in people with advanced dementia. BMC Geriatr. 22 (1), 214. 10.1186/s12877-022-02914-x 35296254 PMC8925050

[B151] Ries M. Sastre M. (2016). Mechanisms of Aβ clearance and degradation by glial cells. Front. Aging Neurosci. 8, 160. 10.3389/fnagi.2016.00160 27458370 PMC4932097

[B152] Rodriguez-Giraldo M. Gonzalez-Reyes R. E. Ramirez-Guerrero S. Bonilla-Trilleras C. E. Guardo-Maya S. Nava-Mesa M. O. (2022). Astrocytes as a therapeutic target in Alzheimer's disease-comprehensive review and recent developments. Int. J. Mol. Sci. 23 (21), 13630. 10.3390/ijms232113630 36362415 PMC9654484

[B153] Romermann K. Fedrowitz M. Hampel P. Kaczmarek E. Tollner K. Erker T. (2017). Multiple blood-brain barrier transport mechanisms limit bumetanide accumulation, and therapeutic potential, in the mammalian brain. Neuropharmacology 117, 182–194. 10.1016/j.neuropharm.2017.02.006 28192112

[B154] Ronaldson P. T. Williams E. I. Betterton R. D. Stanton J. A. Nilles K. L. Davis T. P. (2024). CNS drug delivery in stroke: improving therapeutic translation from the bench to the bedside. Stroke 55 (1), 190–202. 10.1161/strokeaha.123.043764 38134249 PMC10752297

[B155] Rost N. S. Brodtmann A. Pase M. P. van Veluw S. J. Biffi A. Duering M. (2022). Post-stroke cognitive impairment and dementia. Circ. Res. 130 (8), 1252–1271. 10.1161/CIRCRESAHA.122.319951 35420911

[B156] Ruocco A. Nicole O. Docagne F. Ali C. Chazalviel L. Komesli S. (1999). A transforming growth factor-beta antagonist unmasks the neuroprotective role of this endogenous cytokine in excitotoxic and ischemic brain injury. J. Cereb. Blood Flow. Metab. 19 (12), 1345–1353. 10.1097/00004647-199912000-00008 10598939

[B157] Salloway S. Chalkias S. Barkhof F. Burkett P. Barakos J. Purcell D. (2022). Amyloid-related imaging abnormalities in 2 phase 3 studies evaluating aducanumab in patients with early alzheimer disease. JAMA Neurol. 79 (1), 13–21. 10.1001/jamaneurol.2021.4161 34807243 PMC8609465

[B158] Salvadori E. Poggesi A. Donnini I. Rinnoci V. Chiti G. Squitieri M. (2021). Efficacy and safety of the association of nimodipine and choline alphoscerate in the treatment of cognitive impairment in patients with cerebral small vessel disease. The CONIVaD trial. Drugs Aging 38 (6), 481–491. 10.1007/s40266-021-00852-8 33855653 PMC8211589

[B159] Savardi A. Borgogno M. De Vivo M. Cancedda L. (2021). Pharmacological tools to target NKCC1 in brain disorders. Trends Pharmacol. Sci. 42 (12), 1009–1034. 10.1016/j.tips.2021.09.005 34620512

[B160] Schaub G. A. Schnitker A. (1988). Influence of Blastocrithidia triatomae (Trypanosomatidae) on the reduviid bug *Triatoma infestans*: alterations in the Malpighian tubules. Parasitol. Res. 75 (2), 88–97. 10.1007/bf00932706 3148931

[B161] Sevigny J. Chiao P. Bussiere T. Weinreb P. H. Williams L. Maier M. (2016). The antibody aducanumab reduces Aβ plaques in Alzheimer's disease. Nature 537 (7618), 50–56. 10.1038/nature19323 27582220

[B162] Sheng Z. Liu Y. Li H. Zheng W. Xia B. Zhang X. (2018). Efficacy of minocycline in acute ischemic stroke: a systematic review and meta-analysis of rodent and clinical studies. Front. Neurol. 9, 1103. 10.3389/fneur.2018.01103 30619060 PMC6306456

[B163] Shigetomi E. Jackson-Weaver O. Huckstepp R. T. O'Dell T. J. Khakh B. S. (2013). TRPA1 channels are regulators of astrocyte basal calcium levels and long-term potentiation via constitutive D-serine release. J. Neurosci. 33 (24), 10143–10153. 10.1523/JNEUROSCI.5779-12.2013 23761909 PMC3682388

[B164] Sica D. A. (2004). Diuretic-related side effects: development and treatment. J. Clin. Hypertens. (Greenwich) 6 (9), 532–540. 10.1111/j.1524-6175.2004.03789.x 15365284 PMC8109680

[B165] Simpson I. A. Carruthers A. Vannucci S. J. (2007). Supply and demand in cerebral energy metabolism: the role of nutrient transporters. J. Cereb. Blood Flow. Metab. 27 (11), 1766–1791. 10.1038/sj.jcbfm.9600521 17579656 PMC2094104

[B166] Sivakumaran S. Maguire J. (2016). Bumetanide reduces seizure progression and the development of pharmacoresistant status epilepticus. Epilepsia 57 (2), 222–232. 10.1111/epi.13270 26659482 PMC5487491

[B167] Snyder H. M. Corriveau R. A. Craft S. Faber J. E. Greenberg S. M. Knopman D. (2015). Vascular contributions to cognitive impairment and dementia including Alzheimer's disease. Alzheimers Dement. 11 (6), 710–717. 10.1016/j.jalz.2014.10.008 25510382 PMC4731036

[B168] Sofroniew M. V. (2015). Astrocyte barriers to neurotoxic inflammation. Nat. Rev. Neurosci. 16 (5), 249–263. 10.1038/nrn3898 25891508 PMC5253239

[B169] Sofroniew M. V. (2020). Astrocyte reactivity: subtypes, states, and functions in CNS innate immunity. Trends Immunol. 41 (9), 758–770. 10.1016/j.it.2020.07.004 32819810 PMC7484257

[B170] Song S. Luo L. Sun B. Sun D. (2019). Roles of glial ion transporters in brain diseases. Glia 68, 472–494. 10.1002/glia.23699 31418931 PMC6957693

[B171] Song S. Wang S. Pigott V. M. Jiang T. Foley L. M. Mishra A. (2018). Selective role of Na(+)/H(+) exchanger in Cx3cr1(+) microglial activation, white matter demyelination, and post-stroke function recovery. Glia 66 (11), 2279–2298. 10.1002/glia.23456 30043461 PMC6430713

[B172] Sperling R. A. Donohue M. C. Raman R. Rafii M. S. Johnson K. Masters C. L. (2023). Trial of solanezumab in preclinical Alzheimer's disease. N. Engl. J. Med. 389 (12), 1096–1107. 10.1056/NEJMoa2305032 37458272 PMC10559996

[B173] Sperling R. A. Jack C. R. Jr. Black S. E. Frosch M. P. Greenberg S. M. Hyman B. T. (2011). Amyloid-related imaging abnormalities in amyloid-modifying therapeutic trials: recommendations from the Alzheimer's Association Research Roundtable Workgroup. Alzheimers Dement. 7 (4), 367–385. 10.1016/j.jalz.2011.05.2351 21784348 PMC3693547

[B174] Su F. Bai F. Zhang Z. (2016). Inflammatory cytokines and Alzheimer's disease: a review from the perspective of genetic polymorphisms. Neurosci. Bull. 32 (5), 469–480. 10.1007/s12264-016-0055-4 27568024 PMC5563762

[B175] Su G. Kintner D. B. Flagella M. Shull G. E. Sun D. (2002). Astrocytes from Na(+)-K(+)-Cl(-) cotransporter-null mice exhibit absence of swelling and decrease in EAA release. Am. J. Physiol. Cell Physiol. 282 (5), C1147–C1160. 10.1152/ajpcell.00538.2001 11940530

[B176] Taubes A. Nova P. Zalocusky K. A. Kosti I. Bicak M. Zilberter M. Y. (2021). Experimental and real-world evidence supporting the computational repurposing of bumetanide for APOE4-related Alzheimer’s disease. Nat. Aging 1 (10), 932–947. 10.1038/s43587-021-00122-7 36172600 PMC9514594

[B177] Tayebati S. K. Di Tullio M. A. Tomassoni D. Amenta F. (2009). Neuroprotective effect of treatment with galantamine and choline alphoscerate on brain microanatomy in spontaneously hypertensive rats. J. Neurol. Sci. 283 (1-2), 187–194. 10.1016/j.jns.2009.02.349 19304299

[B178] Thacker E. L. McKnight B. Psaty B. M. Longstreth W. T. Jr. Sitlani C. M. Dublin S. (2013). Atrial fibrillation and cognitive decline: a longitudinal cohort study. Neurology 81 (2), 119–125. 10.1212/WNL.0b013e31829a33d1 23739229 PMC3770176

[B179] Thiel A. Cechetto D. F. Heiss W. D. Hachinski V. Whitehead S. N. (2014). Amyloid burden, neuroinflammation, and links to cognitive decline after ischemic stroke. Stroke 45 (9), 2825–2829. 10.1161/STROKEAHA.114.004285 25005439

[B180] Tollner K. Brandt C. Topfer M. Brunhofer G. Erker T. Gabriel M. (2014). A novel prodrug-based strategy to increase effects of bumetanide in epilepsy. Ann. Neurol. 75 (4), 550–562. 10.1002/ana.24124 24615913

[B181] Traiffort E. Kassoussi A. Zahaf A. Laouarem Y. (2020). Astrocytes and microglia as major players of myelin production in normal and pathological conditions. Front. Cell Neurosci. 14, 79. 10.3389/fncel.2020.00079 32317939 PMC7155218

[B182] van Dyck C. H. Swanson C. J. Aisen P. Bateman R. J. Chen C. Gee M. (2023). Lecanemab in early Alzheimer's disease. N. Engl. J. Med. 388 (1), 9–21. 10.1056/NEJMoa2212948 36449413

[B183] Vitari A. C. Thastrup J. Rafiqi F. H. Deak M. Morrice N. A. Karlsson H. K. (2006). Functional interactions of the SPAK/OSR1 kinases with their upstream activator WNK1 and downstream substrate NKCC1. Biochem. J. 397 (1), 223–231. 10.1042/BJ20060220 16669787 PMC1479760

[B184] Wang J. Cheng C. Liu Z. Lin Y. Yang L. Zhang Z. (2023). Inhibition of A1 astrocytes and activation of A2 astrocytes for the treatment of spinal cord injury. Neurochem. Res. 48 (3), 767–780. 10.1007/s11064-022-03820-9 36418652

[B185] Wang J. Zhang H. Y. Tang X. C. (2009). Cholinergic deficiency involved in vascular dementia: possible mechanism and strategy of treatment. Acta Pharmacol. Sin. 30 (7), 879–888. 10.1038/aps.2009.82 19574993 PMC4006646

[B186] Wang S. Wang B. Shang D. Zhang K. Yan X. Zhang X. (2022). Ion Channel dysfunction in astrocytes in neurodegenerative diseases. Front. Physiol. 13, 814285. 10.3389/fphys.2022.814285 35222082 PMC8864228

[B187] Weyand C. M. Goronzy J. J. (2016). Aging of the immune system. Mechanisms and therapeutic targets. Ann. Am. Thorac. Soc. 13 (5), S422–s428. 10.1513/AnnalsATS.201602-095AW 28005419 PMC5291468

[B188] WHO (2017). Global action plan on the public health response to dementia 2017–2025 . https://www.who.int/publications/i/item/global-action-plan-on-the-public-health-response-to-dementia-2017---2025 (Accessed January 20, 2024).

[B189] WHO (2021). World failing to address dementia challenge . https://www.who.int/news/item/02-09-2021-world-failing-to-address-dementia-challenge (Accessed March 18, 2024).

[B190] WHO (2023). Dementia . https://www.who.int/news-room/fact-sheets/detail/dementia (Accessed March 18, 2024).

[B191] Willis B. A. Sundell K. Lachno D. R. Ferguson-Sells L. R. Case M. G. Holdridge K. (2018). Central pharmacodynamic activity of solanezumab in mild Alzheimer's disease dementia. Alzheimers Dement. (N Y) 4, 652–660. 10.1016/j.trci.2018.10.001 30511011 PMC6258891

[B192] Withington C. G. Turner R. S. (2022). Amyloid-related imaging abnormalities with anti-amyloid antibodies for the treatment of dementia due to Alzheimer's disease. Front. Neurol. 13, 862369. 10.3389/fneur.2022.862369 35401412 PMC8985815

[B193] Wong L. J. Lee B. W. L. Sng Y. J. Poh L. Rajeev V. Selvaraji S. (2023). Inflammasome activation mediates apoptotic and pyroptotic death in astrocytes under ischemic conditions. Neuromolecular Med. 25 (4), 533–544. 10.1007/s12017-023-08753-2 37646911

[B194] Wu T. Dejanovic B. Gandham V. D. Gogineni A. Edmonds R. Schauer S. (2019). Complement C3 is activated in human AD brain and is required for neurodegeneration in mouse models of amyloidosis and tauopathy. Cell Rep. 28 (8), 2111–2123. 10.1016/j.celrep.2019.07.060 31433986

[B195] Wu X. Gao Y. Shi C. Tong J. Ma D. Shen J. (2023). Complement C1q drives microglia-dependent synaptic loss and cognitive impairments in a mouse model of lipopolysaccharide-induced neuroinflammation. Neuropharmacology 237, 109646. 10.1016/j.neuropharm.2023.109646 37356797

[B196] Xiao T. Ji H. Shangguan X. Qu S. Cui Y. Xu J. (2022). NLRP3 inflammasome of microglia promotes A1 astrocyte transformation, neo-neuron decline and cognition impairment in endotoxemia. Biochem. Biophys. Res. Commun. 602, 1–7. 10.1016/j.bbrc.2022.02.092 35247698

[B197] Xu Y. Yang Y. Chen X. Jiang D. Zhang F. Guo Y. (2023). NLRP3 inflammasome in cognitive impairment and pharmacological properties of its inhibitors. Transl. Neurodegener. 12 (1), 49. 10.1186/s40035-023-00381-x 37915104 PMC10621314

[B198] Xu H H. Garcia-Ptacek S. Jönsson L. Wimo A. Nordström P. Eriksdotter M. (2021). Long-term effects of cholinesterase inhibitors on cognitive decline and mortality. Neurology 96 (17), e2220–e2230. 10.1212/wnl.0000000000011832 33741639 PMC8166426

[B199] Xu J J. J. Guo S. Xue R. Xiao L. Kou J. N. Liu Y. Q. (2021). Adalimumab ameliorates memory impairments and neuroinflammation in chronic cerebral hypoperfusion rats. Aging (Albany NY) 13 (10), 14001–14014. 10.18632/aging.203009 34030135 PMC8202885

[B200] Yamada K. Park H. M. Rigel D. F. DiPetrillo K. Whalen E. J. Anisowicz A. (2016). Small-molecule WNK inhibition regulates cardiovascular and renal function. Nat. Chem. Biol. 12 (11), 896–898. 10.1038/nchembio.2168 27595330

[B201] Yin J. Zhao F. Chojnacki J. E. Fulp J. Klein W. L. Zhang S. (2018). NLRP3 inflammasome inhibitor ameliorates amyloid pathology in a mouse model of Alzheimer's disease. Mol. Neurobiol. 55 (3), 1977–1987. 10.1007/s12035-017-0467-9 28255908 PMC5585057

[B202] Yu Y. Fu P. Yu Z. Xie M. Wang W. Luo X. (2018). NKCC1 inhibition attenuates chronic cerebral hypoperfusion-induced white matter lesions by enhancing progenitor cells of oligodendrocyte proliferation. J. Mol. Neurosci. 64 (3), 449–458. 10.1007/s12031-018-1043-0 29502291

[B203] Yun S. P. Kam T. I. Panicker N. Kim S. Oh Y. Park J. S. (2018). Block of A1 astrocyte conversion by microglia is neuroprotective in models of Parkinson's disease. Nat. Med. 24 (7), 931–938. 10.1038/s41591-018-0051-5 29892066 PMC6039259

[B204] Zamanian J. L. Xu L. Foo L. C. Nouri N. Zhou L. Giffard R. G. (2012). Genomic analysis of reactive astrogliosis. J. Neurosci. 32 (18), 6391–6410. 10.1523/JNEUROSCI.6221-11.2012 22553043 PMC3480225

[B205] Zhang J. Bhuiyan M. I. H. Zhang T. Karimy J. K. Wu Z. Fiesler V. M. (2020). Modulation of brain cation-Cl(-) cotransport via the SPAK kinase inhibitor ZT-1a. Nat. Commun. 11 (1), 78. 10.1038/s41467-019-13851-6 31911626 PMC6946680

[B206] Zhang Q. Liu C. Shi R. Zhou S. Shan H. Deng L. (2022). Blocking C3d(+)/GFAP(+) A1 astrocyte conversion with Semaglutide attenuates blood-brain barrier disruption in mice after ischemic stroke. Aging Dis. 13 (3), 943–959. 10.14336/AD.2021.1029 35656116 PMC9116904

[B207] Zhang R. Wu Y. Xie F. Zhong Y. Wang Y. Xu M. (2018). RGMa mediates reactive astrogliosis and glial scar formation through TGFβ1/Smad2/3 signaling after stroke. Cell Death Differ. 25 (8), 1503–1516. 10.1038/s41418-018-0058-y 29396549 PMC6113216

[B208] Zhao H. Nepomuceno R. Gao X. Foley L. M. Wang S. Begum G. (2017). Deletion of the WNK3-SPAK kinase complex in mice improves radiographic and clinical outcomes in malignant cerebral edema after ischemic stroke. J. Cereb. Blood Flow. Metab. 37 (2), 550–563. 10.1177/0271678X16631561 26861815 PMC5381450

[B209] Zhou Z. Zhan J. Cai Q. Xu F. Chai R. Lam K. (2022). The water transport system in astrocytes-aquaporins. Cells 11 (16), 2564. 10.3390/cells11162564 36010640 PMC9406552

[B210] Zlokovic B. V. Gottesman R. F. Bernstein K. E. Seshadri S. McKee A. Snyder H. (2020). Vascular contributions to cognitive impairment and dementia (VCID): a report from the 2018 national heart, Lung, and blood Institute and national Institute of neurological disorders and stroke workshop. Alzheimers Dement. 16 (12), 1714–1733. 10.1002/alz.12157 33030307

